# The parabrachial to central amygdala pathway is critical to injury-induced pain sensitization in mice

**DOI:** 10.1038/s41386-023-01673-6

**Published:** 2023-08-04

**Authors:** Jeitzel M. Torres-Rodriguez, Torri D. Wilson, Sudhuman Singh, Maria L. Torruella-Suárez, Sarah Chaudhry, Anisha P. Adke, Jordan J. Becker, Benjamin Neugebauer, Jenny L. Lin, Santiago Martinez Gonzalez, Omar Soler-Cedeño, Yarimar Carrasquillo

**Affiliations:** 1 National Center for Complementary and Integrative Health, National Institutes of Health, Bethesda, MD USA; 2 National Institute on Drug Abuse, National Institutes of Health, Bethesda, MD USA

**Keywords:** Neuroscience, Cellular neuroscience

## Abstract

The spino-ponto-amygdaloid pathway is a major ascending circuit relaying nociceptive information from the spinal cord to the brain. Potentiation of excitatory synaptic transmission in the parabrachial nucleus (PBN) to central amygdala (CeA) pathway has been reported in rodent models of persistent pain. However, the functional significance of this pathway in the modulation of the somatosensory component of pain was recently challenged by studies showing that spinal nociceptive neurons do not target CeA-projecting PBN cells and that manipulations of this pathway have no effect on reflexive-defensive somatosensory responses to peripheral noxious stimulation. Here, we showed that activation of CeA-projecting PBN neurons is critical to increase both stimulus-evoked and spontaneous nociceptive responses following an injury in male and female mice. Using optogenetic-assisted circuit mapping, we confirmed a functional excitatory projection from PBN→CeA that is independent of the genetic or firing identity of CeA cells. We then showed that peripheral noxious stimulation increased the expression of the neuronal activity marker Fos in CeA-projecting PBN neurons and that chemogenetic inactivation of these cells decreased behavioral hypersensitivity in models of neuropathic and inflammatory pain without affecting baseline nociception. Lastly, we showed that chemogenetic activation of CeA-projecting PBN neurons is sufficient to induced bilateral hypersensitivity without injury. Together, our results indicate that the PBN→CeA pathway is a key modulator of pain-related behaviors that can increase reflexive-defensive and affective-motivational responses to somatosensory stimulation in injured states without affecting nociception under normal physiological conditions.

## Introduction

Chronic pain is a multidimensional experience that encompasses reflexive-defensive somatosensory and affective-motivational components. In the United States alone, more than 1 in 5 Americans suffer from chronic pain [1], with similar statistics observed across 52 countries [2]. Despite this, diagnostic tools and currently available treatments are limited and can lead to opioid addiction in some people [3]. This state-of-affairs underscore the importance of identifying mechanisms involved in pain processing to potentially improve diagnosis and develop prospective treatments.

The spino-ponto-amygdaloid pathway is a major ascending pathway involved in the relay of nociceptive information from the spinal cord to the brain [4–7]. In this pathway, peripheral nociceptors receive and relay nociceptive inputs to second order neurons in lamina I of the spinal cord, which then send projections to the pontine parabrachial nucleus (PBN) [8–11]. Several studies have shown that PBN neurons respond to noxious stimuli [8, 12–14] and function as a hub for the relay of nociceptive information to multiple brain regions, including the periaqueductal gray, hypothalamic and thalamic nuclei, and extended amygdala nuclei [12, 15–17].

Among these brain areas, the central amygdala (CeA) is anatomically well-positioned to integrate somatosensory and affective signals within the brain. It receives somatosensory signals via the spino-ponto-amygdaloid pain pathway and polymodal information, including those related to affective and cognitive states, via inputs from the basolateral and lateral amygdala nuclei, which in turn receive inputs from cortical and thalamic regions [18]. In-vivo and ex-vivo studies have shown that CeA neurons respond to peripheral noxious stimuli and are sensitized following injury in several rodent models of pain [19–21]. At the behavioral level, manipulations of CeA neurons have been shown to modulate reflexive-defensive and affective-motivational components of pain in response to injury and to contribute to different types of analgesia [22–26].

The proposed function of the PBN→CeA pathway in pain processing is supported by ex-vivo electrophysiological studies that have shown potentiation of glutamatergic synaptic transmission in response to injury [20, 21, 27–30]. Consistently, behavioral studies showed that CeA-projecting PBN neurons modulate escape behaviors, affective-motivational responses to painful stimuli, aversion, and threat memory [12, 31, 32]. A notable finding from these studies is that reflexive-defensive responses to noxious stimuli are unaltered by manipulations of this pathway [12, 31–33, 35]. These combined results suggest that the PBN→CeA pathway contributes to the affective-motivational but not the somatosensory component of pain. This notion was further supported by studies showing that spinal nociceptive neurons do not target CeA-projecting PBN neurons [34–36]. In the present study, we determined the function of the PBN→CeA pathway in baseline nociception and injury-induced peripheral hypersensitivity using mouse models of inflammatory and neuropathic pain coupled with viral-mediated anatomical tracers, optogenetic-assisted circuit mapping, and intersectional chemogenetic methods. Our results indicate that the PBN→CeA pathway is critical to increase both stimulus-evoked and spontaneous nociceptive responses in injured states, without altering baseline nociception.

## Materials and methods

### Subjects

Experiments were approved by the Animal Care and Use Committee of the National Institute of Neurological Disorders and Stroke and the National Institute on Deafness and other Communication Disorders with the guidance from the National Institutes of Health (NIH). In-house bred or purchased (Jackson Laboratory) C57BL/6J or Swiss Webster mice aged 8–17 weeks were used for all behavioral and histological experiments. Electrophysiological experiments were performed using heterozygous *Prkcd*-cre mice (GENSAT – founder line 011559-UCD) or heterozygous *Sst*-cre (Jackson Laboratory – founder line 018973) crossed with homozygous Ai9 mice (Jackson Laboratories – founder line 007909). The expression and fidelity of Cre in Som+ and PKCδ+ neurons have been previously described [23, 37]. Details about genotyping, housing conditions and handling are described in Supplementary information. Behavioral tests were conducted under red light during the dark period (10 a.m. to 6 p.m.). The sex of the mice used for each experiment is described in the Supplementary information and figure legends. Animals were randomly assigned to experimental groups and all experiments and analyses were performed blinded to experimental treatment.

### Stereotaxic injections

Stereotaxic surgeries for acute injections into the CeA and PBN were performed using standard procedures as described previously [23]. Detailed description of viruses, coordinates and procedures for injection site verification used for each experiment are described in Supplementary information.

### Sciatic cuff implantation and nociceptive testing

Surgeries for cuff implantation were performed as described previously [23, 38, 39]. Nociceptive testing on males and females was performed separately. Experimenter was blind to treatment for all behavioral testing and every cohort was counterbalanced to include mice from all experimental groups. von Frey, acetone, Hargreaves, and Randall-Selitto were used to measure sensitivity to tactile, cold, heat and deep tissue pressure, respectively, using standard procedures described previously [22, 40–42]. The formalin test was used as model inflammatory pain as described previously [43–45]. Detailed description for cuff surgeries and behavioral testing, including timelines for each experiment, are included in Supplementary information.

### Immunohistochemistry, image acquisition and analysis

Standard procedures were used for all histological experiments as described previously [23]. A detailed description of experimental timelines, antibodies, anatomical definitions of individual brain regions, imaging, quantification, and analyses parameters are included in the Supplementary information.

### Ex-vivo electrophysiology

Preparation of acute brain slices for electrophysiological experiments, whole-cell, patch-clamp recordings in CeA and PBN neurons, optogenetic-assisted circuit mapping and validation of chemogenetic experiments were performed using standard procedures as previously described [23, 46, 47]. Details for experimental procedures, timelines, and analyses of individual experiments are included in Supplementary information.

### Statistical analysis

Data are presented as mean ± SEM. Statistical analysis was performed using unpaired or paired two tailed t test, or two-way analysis of variance (ANOVA) followed by Tukey’s multiple comparison tests using Graph Pad Prism version 9.0. The significance level was set at *p* < 0.05. Sample sizes and *p* values are described in each figure legend. Detailed information on each statistical test performed are shown in Table S1.

## Results

### Excitatory projection from PBN→CeA is independent of the genetic or firing identity of CeA cells

**Fig. 1:**
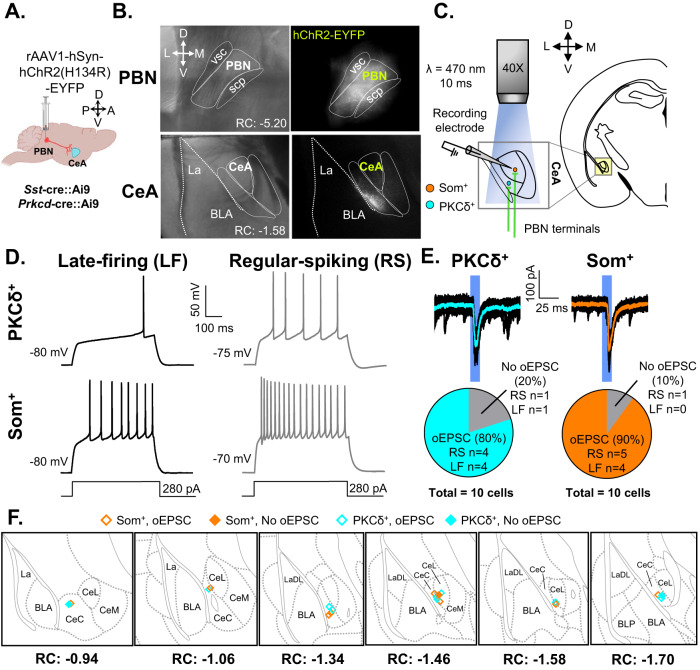
Validation of functional projection from PBN to CeA. **A** Male Som-Cre::Ai9 and PKCδ::Ai9 mice were stereotaxically injected with rAAV1-hSyn-hChR2(H134R)-EYFP into the PBN. Whole-cell patch-clamp recordings were performed on mice at least 4 weeks following the injection. **B** Representative images of the PBN (top) and CeA (bottom). Left panel depicts differential interference contrast images. Right panel depicts fluorescent images of transduced cells within the PBN and fluorescent PBN terminals within the CeA. **C** Schematic depicting optogenetic-assisted circuit mapping experiments. Whole-cell patch-clamp recordings of optically evoked excitatory postsynaptic current (oEPSC) in response to blue light (470 nm) stimulation of PBN terminals in CeA-Som+ or CeA-PKCδ+ neurons. **D** Representative voltage traces of late-firing (LF; left panel) and regular-spiking (RS; right panel) CeA-PKCδ+ (top) and CeA-Som+ (bottom) neurons in response to a 280-pA depolarizing current injection. **E** Representative current traces of oEPSCs in CeA-PKCδ+ and CeA-Som+ neurons in response to a 10 ms pulse of blue light stimulation. The proportion of CeA-PKCδ+ LF and RS neurons displaying oEPSCs was comparable to the proportion of CeA-Som+ LF and RS neurons displaying oEPSCs. *n* = 10 cells collected from 2 mice per genotype. No intra-animal correlations were seen. **F** Recording site illustrations of CeA-Som+ and CeA-PKCδ+ neurons classified by either the presence or absence of oEPSCs. Each symbol highlights the location of an individual neuron. vsc ventral spinocerebellar tract, scp superior cerebellar peduncle, PBN parabrachial nucleus, CeA central amygdala, CeM central amygdala medial, CeL central amygdala lateral, CeC central amygdala capsular, BLA basolateral amygdaloid, LA lateral amygdaloid.

To validate the functional circuitry between the PBN and CeA, we used optogenetic-assisted circuit mapping in acute brain slices from *Sst*-cre::Ai9 or *Prkcd*-cre::Ai9 mice injected with AAV-hChR2-EYFP into the PBN (Fig. 1A–C). Consistent with previous reports [4, 27] fluorescent terminals were readily observed within the laterocapsular subdivision of the CeA (CeLC) when the Channelrhodopsin-2 (ChR2)-expressing virus was injected into the PBN (Fig. 1B). Additionally, firing phenotypes in PKCδ+ and Som+ CeA neurons were heterogeneous [46, 48, 49] (Fig. 1D). Blue light stimulation of PBN terminals in the CeA evoked excitatory postsynaptic currents (oEPSCs) in the majority of PKCδ+ (80%) and Som+ neurons (90%) recorded (Fig. 1E, F). These results were obtained regardless of firing phenotype (Fig. 1E). Together, these findings confirm that CeLC neurons receive excitatory efferent projections originating from the PBN independently of the genetic or firing identity of CeA neurons.

### Peripheral noxious stimulation induced Fos expression in CeA-projecting PBN neurons without affecting intrinsic excitability after nerve injury

Previous studies have claimed that CeA-projecting PBN neurons do not receive direct nociceptive inputs from the spinal cord [32, 35, 36]. However, separate studies have shown that the PBN is activated in response to peripheral noxious stimulation [12, 13, 50, 51] and that injury potentiates the PBN→CeA pathway in several mouse models of persistent pain. Neurons expressing the calcitonin gene-related peptide (CGRP) are among the PBN neurons activated by noxious stimulation [52]. CGRP-expressing PBN neurons have also been shown to project to the CeA and to be anatomically associated with presynaptic terminals of spinoparabrachial neurons [31, 53]. Whether CeA-projecting PBN neurons are activated by peripheral noxious stimulation has not been directly tested.

We addressed this question by measuring Fos in response to pinch stimulation of the hind paw. Fos is a marker of neuronal activity previously shown to peak in the PBN 60–90 min after noxious stimulation that returns to baseline after 8 h [12, 13]. We used an intersectional genetic approach for the identification of CeA-projecting PBN neurons. C57BL/6J mice were injected with a cre-expressing retrograde Adeno-associated virus (AAV) (pENN.AAV.hSyn.HI.eGFP-Cre.WPRE.SV40) into the CeA and a cre-dependent AAV encoding the red fluorescent protein mCherry (AAV8-hSyn-DIO-mcherry) into the PBN (Fig. S1). A pAAV.CMV encoding LacZ was co-injected with the cre-expressing retrograde AAV to identify the injection site in the CeA. As illustrated in Figure S1B–D, LacZ expression was restricted to the CeA, and robust transduction efficacy was observed at all CeA rostro-caudal levels. Evaluation of mCherry expression in the PBN showed robust transduction throughout the rostro-caudal PBN that was mostly restricted to the external lateral PBN (Fig. S1E–G). These results confirm that we can selectively visualize CeA-projecting PBN neurons using this intersectional genetic strategy.

**Fig. 2:**
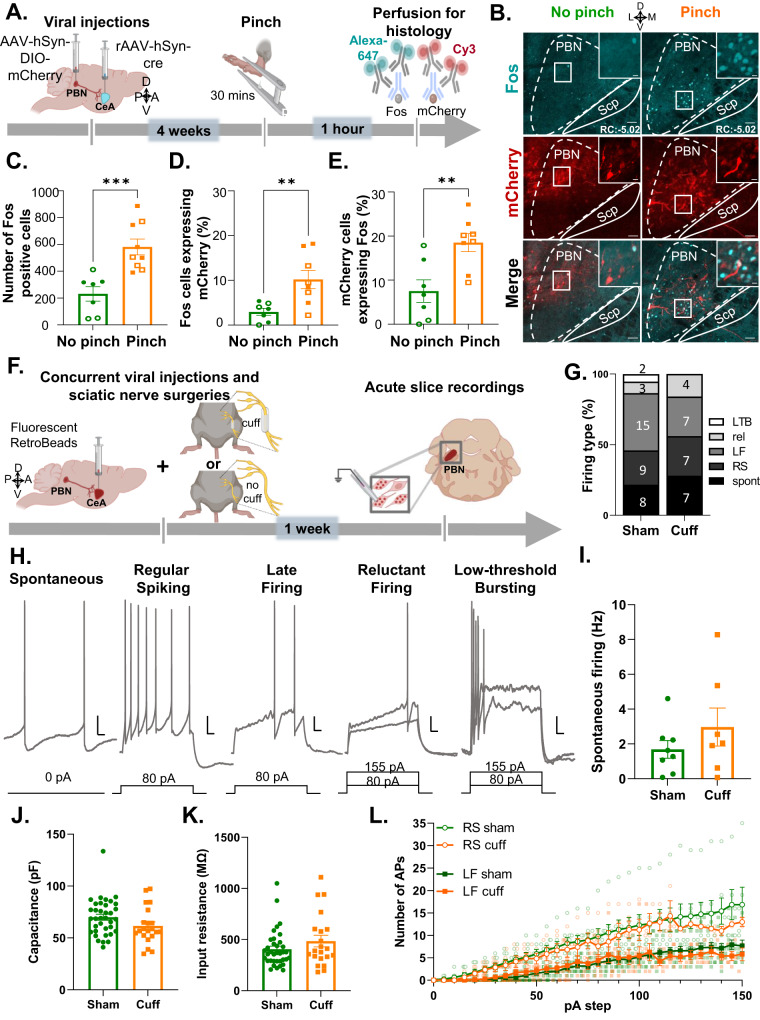
Peripheral noxious stimulation induces Fos expression but intrinsic excitability is unaltered by nerve injury in CeA-projecting PBN neurons. **A** Timeline of experiments. Male and female C57BL/6J mice were stereotaxically injected with AAV8-hSyn-DIO-mCherry into the right PBN and AAV.hSyn.HI.eGFP-Cre into the right CeA. Four weeks after viral injections, we performed bilateral pinch stimulation on the hind paws for 30 min followed by transcardial perfusion for histology. **B** Representative images of right PBN slices immunostained for mCherry and Fos (cyan). No-pinch (left) and pinch (right) panels show low magnification images of Fos (top) and mCherry (middle). High magnification images of merged signals in area delineated by white box are shown in the right panel. Scale bars represent 200 µm for low magnification and 25 µm for high magnification images. Solid arrows represent cells co-labeled with mCherry and Fos while open arrows show Fos only positive cells. Rostro-caudal level relative to bregma (RC) \for both animals is −4.96. **C** Fos+ cells of control (no pinch) vs experimental (pinch) mice in the PBN. *n* = 7 no-pinch mice (3 female and 4 male) and *n* = 9 pinch mice (4 female and 5 male); number of Fos positive cells represents the sum of positive cells in a total of 4 slices from the right PBN (RC levels: −4.96, −5.02, −5.20, −5.34 relative to bregma) per mouse. **D** Percentage of mCherry cells co-labeled with Fos in the PBN of pinch vs no-pinch mice. **E** Percentage of Fos+ cells co-labeled with mCherry in the PBN of pinch vs no-pinch mice. *n* = 7 mice for no-pinch (3 female and 4 male) and *n* = 8 mice for pinch (4 female and 4 male). Unpaired two tailed *t* test; **p < 0.01; ***p < 0.001. Individual mice are represented by scatter points and open symbols represent female mice. All data is presented as means ± SEM. **F** Experimental timeline. Male C57BL/6J mice were concurrently stereotaxically injected with retrobeads into the CeA and implanted with a sciatic nerve cuff. Slice electrophysiology experiments were done 7–10 days after surgery. **G** Relative proportion of firing types was not different between pain groups. Digits inside bars represent the number of cells per firing type. Scale bars represent 10 mV and 50 ms for vertical and horizontal scales, respectively. **H** Firing types of CeA-projecting PBN neurons include: spontaneously-firing (spont), regular spiking (RS), late firing (LF), reluctant (rel) and low-threshold bursting (LTB) neurons. Spontaneous firing frequencies (**I**) Capacitance (**J**), input resistance (**K**), and evoked repetitive firing (**L**) are not significantly different between neurons from sham (*n* = 36 cells from 14 mice) and cuff (*n* = 19–20 cells from 12 mice) mice. Unpaired t test (**I**–**K**) and mixed-effects model (REML) with matching (**L**).

Consistent with previous studies [12, 13], evaluation of Fos expression in the PBN of male and female mice showed significant (*p* < 0.001) increases in Fos+ neurons in response to pinch stimulation of the hind paw, when compared to control mice that did not receive pinch stimulation (Fig. 2A–C). Quantification of mCherry+ CeA-projecting PBN neurons co-expressing Fos further showed that approximately 10% of pinch-induced Fos was localized to CeA-projecting PBN neurons (Fig. 2D) and approximately 20% of CeA-projecting PBN neurons expressed Fos after pinching (Fig. 2E).

In contrast, significantly (*p* < 0.05) lower co-expression of Fos was seen in CeA-projecting PBN neurons in control no-pinch conditions (Fig. 2D, E). No significant sex differences in number of positive cells were observed in any of the groups evaluated (Table S1).

Follow-up patch-clamp electrophysiological experiments in acute brain slices showed that CeA-projecting PBN neurons have heterogeneous firing types, including spontaneous, regular spiking, late firing, reluctant firing, and low-threshold bursting neurons (Fig. 2F–H). Evaluation of intrinsic membrane and firing properties following nerve injury further revealed that CeA-projecting PBN neurons are unaltered by sciatic nerve cuff implantation, compared to cells from sham conditions (Fig. 2I–L).

Together with the histological data, these results show that CeA-projecting PBN neurons are activated by peripheral noxious stimulation, but that injury does not lead to plasticity of intrinsic excitability in these PBN cells.

### Chemogenetic inhibition of CeA-projecting PBN neurons decreased cuff-induced hypersensitivity without affecting baseline nociception

To test for a causal relationship between the activity of CeA-projecting PBN neurons and injury-induced hypersensitivity, we used an intersectional chemogenetic approach where we stereotaxically co-injected a cre-expressing retrograde AAV into the CeA and an AAV encoding the cre-dependent inhibitory designer receptor exclusively activated by designer drugs (DREADD) hM4Di into the PBN (Fig. S2A–E). To validate our intersectional chemogenetic approach, we prepared acute brain slices and performed whole-cell current-clamp recordings in hMD4i-transduced PBN neurons before and after bath-administered clozapine-N-oxide (CNO; 10 μM) or saline control (Fig. S2B). As expected, bath-administered CNO, but not saline, significantly (*p* < 0.001) decreased neuronal firing in hM4Di-transduced cells (Fig. S2C), confirming CNO-mediated inhibition of CeA-projecting PBN neurons.

To assess sensitivity to cold, heat, tactile, and deep tissue pressure stimulation, we used the cuff model of neuropathic pain [39] coupled with acetone, Hargreaves, von Frey filaments, and Randall-Selitto tests, respectively (Fig. S2G–J). Sham sciatic nerve surgeries were used as a control to rule out potential surgery-related non-neuropathic effects. Consistent with previous studies [39], sciatic cuff implantation induced robust hypersensitivity in the ipsilateral paw in all four modalities tested (Fig. S2G–J). Thus, compared to sham mice, cuff mice exhibited significantly *(p* < 0.0001) lower withdrawal thresholds to tactile and deep tissue pressure stimulation, higher response scores to cold stimulation, and lower withdrawal latencies to heat stimulation of the hind paw ipsilateral to nerve treatment. Notably, in all modalities tested, bilateral responses to peripheral stimulation in sham animals were comparable to responses of the uninjured paw contralateral to cuff implantation. It is also important to note that in the Randall-Selitto test all mice responded below the cutoff of 200 g (Fig. S2H). Coupled with the small inter-individual variability seen in this test (SEM < 1.8 g), these results indicate consistent levels of anesthesia between mice in this test. Together, these results demonstrate that the sciatic nerve surgical procedure does not affect behavioral responses and that hypersensitivity is restricted to the ipsilateral paw of cuff mice.

**Fig. 3:**
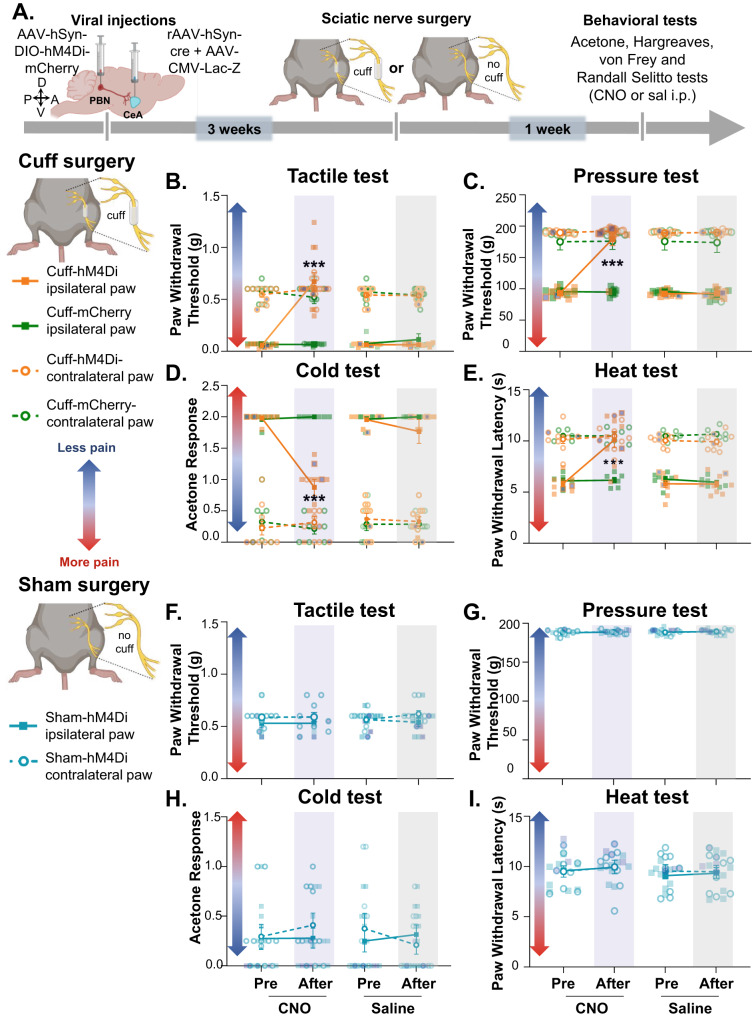
Chemogenetic inhibition of CeA-projecting PBN neurons reverses nerve injury-induced hypersensitivity without affecting baseline nociception. **A** Experimental timeline. Male and female C57BL/6J mice were stereotaxically co-injected with AAV8-hSyn-DIO-hMD4i or AAV8-hSyn-DIO into the right PBN and a mix (1:1) of AAV.hSyn.HI.eGFP-Cre and pAAV.CMV.LacZ.bGH into the right CeA. Sciatic nerve surgery was performed 3 weeks after viral injections. Following 1 week of recovery, von Frey, Randall-Selitto, Acetone and Hargreaves tests were used to address sensitivity to tactile, deep tissue pressure, cold and heat stimulation, respectively, in the hind paws ipsilateral and contralateral to cuff and sham treatment. Mice were intraperitoneally (i.p.) injected with CNO or saline prior to behavior testing in a counterbalanced way. Paw withdrawal threshold in response to tactile (**B,** **F**) or deep tissue pressure (**C,** **G**) stimulation, acetone response score (**D,** **H**) and paw withdrawal latency after heat stimulation (**E,** **I**) of the hind paw ipsilateral or contralateral to cuff (**B–E**) and sham(**F–I**) treatment before and 30 min after i.p. injection of CNO (blue bar) or saline (gray bar). *n* = 10 mice for cuff-hM4Di and sham-hM4di (2 females and 8 males), *n* = 9 mice for cuff-mCherry for both, ipsilateral and contralateral paws in all tests. Two-way repeated measures ANOVA followed by Tukey’s multiple comparisons test; ***p < 0.001 for before and after CNO in the ipsilateral paw of cuff-hMd4i mice; Individual mice are represented by scatter points and female mice are identified in purple. All data are presented as means ± SEM.

Using the cuff neuropathic pain model, we next evaluated the effects of chemogenetic inhibition of CeA-projecting PBN neurons on hypersensitivity to tactile, deep tissue pressure, cold and heat stimulation (Fig. 3A). We found that cuff-induced hypersensitivity to all modalities was reversed by chemogenetic inhibition of CeA-projecting PBN neurons (Fig. 3B–E). Thus, significantly (*p* < 0.001) higher paw withdrawal thresholds to tactile and deep tissue pressure stimulation, lower response scores to acetone, and higher withdrawal latencies to heat stimulation were observed after CNO-mediated chemogenetic inhibition of PBN→CeA neurons, compared to before CNO treatment or control saline-treated mice. No measurable effect was seen after CNO treatment in mice stereotaxically injected in the PBN with the control virus expressing the fluorophore mCherry, indicating that CNO by itself does not alter the nociceptive behaviors evaluated. Responses in the hind paw contralateral to sciatic cuff implantation were also unaltered by chemogenetic inhibition of the PBN→CeA pathway demonstrating that modulation of behavioral responses to noxious stimuli is restricted to injured states.

In contrast, and consistent with previous reports [12, 31, 33], evaluation of control (sham) mice showed that responses to peripheral stimulation were comparable before and after chemogenetic inhibition of CeA-projecting PBN neurons independently of the modality or the hind paw tested (Fig. 3F–I).

Together, these results indicate that activity of CeA-projecting PBN neurons is necessary for hypersensitivity in a model of neuropathic pain but does not modulate baseline responses to cold, heat, tactile and deep tissue pressure stimulation in uninjured states.

### Chemogenetic inhibition of CeA-projecting PBN neurons decreased spontaneous and stimulus-evoked pain-related behaviors after intraplantar formalin injection

**Fig. 4:**
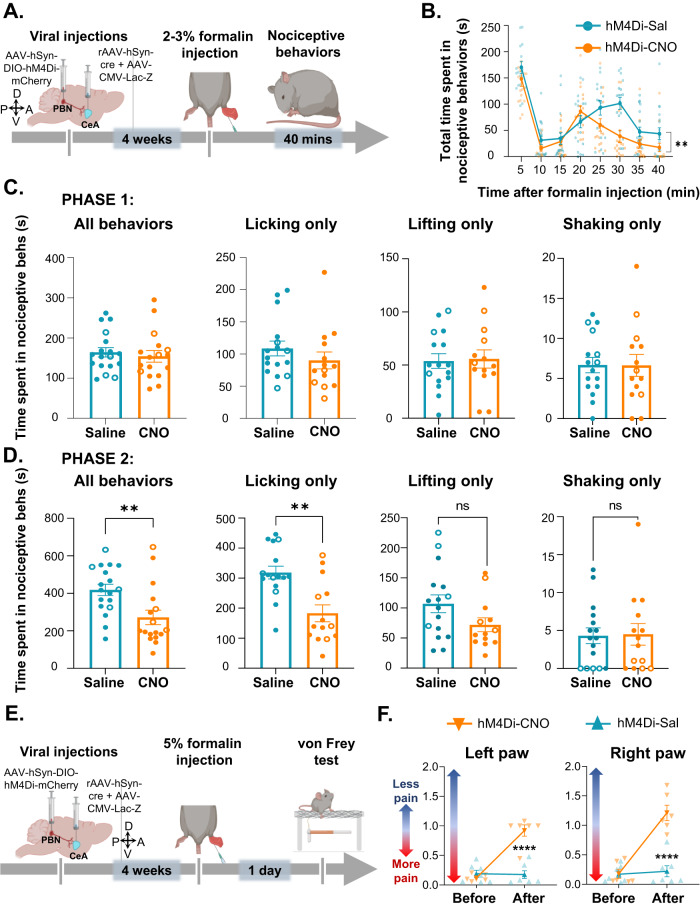
Inhibition of CeA-projecting PBN neurons reduces licking behaviors after intraplantar formalin injection. **A** Timeline of experiments. After 4 weeks of viral injections, mice were injected with 2–3% formalin in the hind paw and nociceptive behaviors were measured for 40 min. **B** Time spent in nociceptive behaviors as a function of time after formalin injection in C57BL/6J mice. *n* = 18 mice (4 females and 14 males per treatment). Two-way repeated measures ANOVA; **p < 0.01. (**C–D**) Time spent in distinct nociceptive behaviors during phase 1 (**C**) and phase 2 (**D**). Phase 1 is defined as 0–5 min post formalin injection and phase 2 as 5–40 min post formalin injection. *n* = 18 mice for saline and *n* = 17 mice for CNO (4 females per treatment). Unpaired two tailed *t* test; **p < 0.01; ***p < 0.001. Individual mice are represented by scatter points and open symbols represent female mice. All data is presented as means ± SEM. **E** Timeline of experiments. After 4 weeks of viral injections, mice were injected with 5% formalin in the hind paw. The von Frey test was performed 1 day after the paw injection in mice that received CNO or saline i.p. injections 30 min before testing. **F** Paw withdrawal threshold in response to tactile stimulation before and after CNO or saline in both hind paws 1 day after formalin injection. *n* = 6 mice for CNO and *n* = 7 mice for saline; two-way repeated measures ANOVA followed by Šídák’s multiple comparisons test; ****p < 0.0001 for before and after CNO. All data is presented as means ± SEM.

Spontaneous pain is among the main complaints in chronic pain patients, and it is reported at higher frequencies than stimulus-evoked pain [54]. In the next experiments we assessed the function of the PBN→CeA pathway in spontaneous nociceptive responses using the formalin model of inflammatory pain. We measured spontaneous nociceptive responses to 2–3% formalin injection into the hind paw of mice following inhibition of CeA-projecting PBN neurons using the intersectional chemogenetic strategy described in the section above (Fig. 4A). Evaluation of the time spent in nociceptive behaviors as a function of time after formalin injection showed a stereotypical biphasic response to formalin in all mice tested [43] (Fig. 4B). However, the time spent in spontaneous nociceptive behaviors during the second phase, was significantly (*p* < 0.05) lower after CNO-mediated chemogenetic inhibition of CeA-projecting PBN neurons than in control saline-treated mice that also received stereotaxic injection of an AAV encoding the hM4Di inhibitory DREADD (Fig. 4B).

Spontaneous nociceptive responses to formalin are defined as lifting, licking, or shaking of the injected hind paw [44, 45]. Recent studies suggested that modulation of distinct behavioral responses to noxious stimuli are circuit specific [55–57]. To determine if modulation of spontaneous nociceptive responses to formalin by CeA-projecting PBN neurons is behavior specific, we analyzed the time spent licking, lifting, or shaking separately in both phase 1 and phase 2 of the formalin test. We found that chemogenetic inhibition of CeA-projecting PBN neurons had no effect in any of the nociceptive responses during phase 1 of the formalin test (Fig. 4C). In contrast, during phase 2, inhibition of CeA-projecting PBN neurons significantly (*p* < 0.01) decreased licking behaviors but not lifting and shaking behaviors (Fig. 4D). We next determined if the PBN→CeA pathway contributes to stimulus-evoked reflexive-defensive responses in the formalin model of inflammatory pain using the von Frey filament test 1d after the paw injection. These experiments showed that CNO-mediated inhibition of these cells significantly (*p* < 0.0001) decreased formalin-induced hypersensitivity to tactile stimulation (Fig. 4E, F). Thus, CNO-treated mice displayed higher paw withdrawal thresholds compared to saline-treated control mice.

Together, our results indicate that activity of CeA-projecting PBN neurons is necessary for formalin-induced spontaneous licking behavioral responses and tactile stimulus-evoked hypersensitivity but that lifting and shaking responses to formalin are not dependent on the PBN→CeA pathway.

### Chemogenetic activation of CeA-projecting PBN neurons induced bilateral hypersensitivity in a modality-specific way

To test whether activation of CeA-projecting PBN neurons is sufficient to induce hypersensitivity to tactile, deep tissue pressure, cold, and heat stimulation, we used an intersectional chemogenetic approach in which we co-injected a cre-expressing retrograde AAV into the CeA and an AAV encoding the cre-dependent excitatory DREADD hM3Dq into the PBN (Fig. S3A). As illustrated in Fig. S3B, the number of transduced CeA-projecting PBN neurons co-expressing the neuronal activity marker. Fos was significantly (*p* < 0.01) higher in brain slices from mice following CNO-induced activation of the hM3Dq DREADD, compared to the number of Fos+ cells in slices from control saline-treated mice. These results confirmed that we can selectively activate CeA-projecting PBN neurons using this intersectional chemogenetic strategy.

**Fig. 5:**
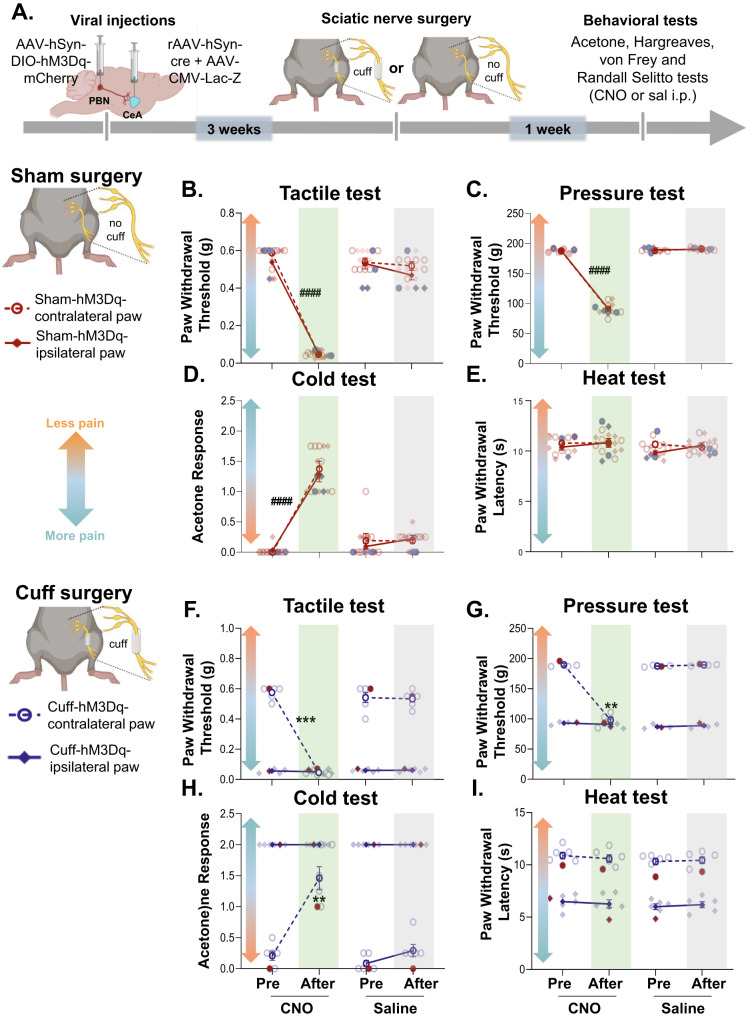
Chemogenetic activation of CeA-projecting PBN neurons induces bilateral hypersensitivity in a modality-specific manner. **A** Experimental timeline of behavioral experiments. Male and female C57BL/6J mice were stereotaxically injected with AAV8-hSyn-DIO-hM3Dq into the right PBN and rAAV-hSyn-cre into the right CeA. Sciatic nerve surgery was performed 3 weeks after viral injections. Following 1 week of recovery, von Frey, Randall-Selitto, Acetone and Hargreaves tests were used to address sensitivity to tactile, deep tissue pressure, cold and heat stimulation, respectively. Mice were intraperitoneally (i.p.) injected with CNO and saline prior to behavior testing in a counterbalanced way. Paw withdrawal threshold after tactile (**B,** **F**) and pinch (**C,** **G**) stimulation, acetone response score (**D,** **H**) and withdrawal latency after heat stimulation (**E,** **I**) of the hind paw ipsilateral or contralateral to sham (**B**–**E**) and cuff (**F**–**I**) treatment before and 30 min after i.p. injection of CNO (green bar) or saline (gray bar). *n* = 7 mice per treatment for sham experiments (2 females and 5 males) and *n* = 6 mice per treatment for cuff experiments (1 female and 5 males). Two-way repeated measures ANOVA followed by Tukey’s multiple comparison test; ^####^*p* < 0.0001 for before and after CNO in contralateral and ipsilateral paws to sham; ****p < 0.0001; **p < 0.01 for before and after CNO in contralateral paw to cuff. Blue (**B**–**E**) and red (**F**–**I**) symbols represent female mice and individual mice are represented by scatter points. All data are presented as means ± SEM.

We then evaluated the effects of chemogenetic activation of CeA-projecting PBN neurons on sensitivity to tactile, deep tissue pressure, cold, and heat stimulation of the hind paw using the von Frey, Randall-Selitto, acetone, and Hargreaves tests, respectively (Fig. 5A). We found that CNO-induced activation of CeA-projecting PBN neurons induced bilateral tactile, deep tissue pressure, and cold hypersensitivity in uninjured mice (Fig. 5B–E). Thus, significantly (*p* < 0.01) lower paw withdrawal thresholds to tactile and deep tissue pressure stimulation and higher acetone response scores were observed following CNO treatment, compared to pretreatment responses and responses in control saline-treated mice. In contrast, paw withdrawal latencies in response to heat stimulation were unaltered by CNO-mediated activation of CeA-projecting PBN neurons (Fig. 5E).

Similar results were observed in the paw contralateral to sciatic nerve cuff implantation, with CNO-treated cuff mice showing significant (*p* < 0.01) hypersensitivity to tactile, deep tissue pressure, and cold, but not heat stimulation, compared to pretreatment responses and responses in control saline-treatment (Fig. 5F–I). No measurable effects were observed in the paw ipsilateral to the cuff implantation after CNO treatment, possibly due to a hypersensitivity ceiling effect. Collectively, our results indicate that activation of CeA-projecting PBN neurons is sufficient to induce bilateral hypersensitivity in the absence of injury in a modality-specific way.

## Discussion

The PBN→CeA is one of the major ascending pain pathways characterized [5–7, 30]. Previous studies have shown that manipulations of PBN neurons in this pathway modulate affective-motivational aspects of pain but not baseline somatosensory responses to peripheral noxious stimulation [12, 31–33]. However, the functionality of this pathway in injury-induced pain sensitization, remains unknown. We showed that CeA-projecting PBN neurons are activated after peripheral noxious stimulation and activity of this pathway is necessary for injury-induced behavioral hypersensitivity, but not baseline nociception. We further showed that chemogenetic activation of this pathway is sufficient to drive hypersensitivity in the absence of injury. These results are consistent with the growing body of evidence supporting the function of the PBN→CeA pathway in pain processing and further demonstrate that this pathway contributes to the amplification of pain-like responses in pathological pain states.

### Activation of CeA-projecting PBN neurons by peripheral noxious stimulation

Our histological experiments showed that a small (~10%) subpopulation of noxious-activated PBN neurons project to the CeA (Fig. 2D, E). Collateral projections to the BNST that modulate aversion have been previously described in CeA-projecting PBN neurons [58]. It is therefore possible that noxious-activated CeA-projecting PBN neurons also project to the BNST and contribute to pain-related aversion. Other studies have further shown that efferent projections from the PBN to the ventromedial nucleus of the hypothalamus, periaqueductal gray, intralaminar thalamic nuclei and the reticular formation modulate escape responses to noxious stimuli [32, 33, 58]. Based on these findings, we predict that Fos+ PBN neurons that do not target the CeA, project to these brain regions and contribute to pain processing.

It is important to note that quantification of pinch-induced Fos was performed in the right PBN following bilateral peripheral stimulation in mice injected with viruses into the right PBN and the right CeA. However, Fos expression in response to noxious stimulation is always bilateral [29]. Despite this, pain-related potentiation of synaptic transmission in the PBN-CeA pathway only occurs in the right hemisphere (right PBN and right CeA) independently of the side of injury [13]. Whether pinch-induced Fos in CeA-projecting PBN neurons is also lateralized, remains unknown.

The results from our Fos experiments were initially surprising given previous reports claiming that spinal nociceptive inputs do not target PBN neurons that project to the CeA [32, 35, 36]. However, classical studies have shown, that injection of a transneuronal retrograde tracer into the CeA results in progressive infection of a multi-synaptic circuit that includes neurons in the parabrachial nucleus, medulla, and dorsal horn [7]. These results show that the dorsal horn of the spinal cord is anatomically linked to PBN neurons that project to the CeA. Consistently, early electron microscopy experiments showed that spinal afferents are synaptically in contact with dendrites of CeA-projecting PBN neurons [5]. CGRP-expressing PBN neurons, known to project to the CeA, have also been shown to be anatomically associated with presynaptic spinal cord terminals [53]. Recent experiments further revealed the existence of a functional microcircuit within the PBN that connects dynorphin-expressing neurons that receive spinal nociceptive inputs with neurons that project to the CeA [12]. The results from our Fos experiments showing activation of the PBN→CeA pathway by peripheral noxious stimulation, coupled with the anatomical and electrophysiological studies described above, suggest that pain-induced activation of the PBN→CeA pathway occurs via excitatory inputs from the dorsal horn of the spinal cord to PBN neurons that project to the CeA.

Our ex-vivo electrophysiological experiments further show that PBN inputs target neurons in the CeLC independently of their genetic or firing identity (Fig. 1). These results are consistent with previous reports showing that most CeLC neurons receive inputs from the PBN [4, 27]. A caveat of these electrophysiological experiments is that Td-tomato expression in the *Sst*-cre::Ai9 and *Prkcd*-cre::Ai9 does not necessarily represent Som and PKCδ expression in the adult tissue used for recordings. Thus, the genetic identity of some of the cells recorded might not be accurate. This concern is mitigated by previous studies confirming that fidelity of expression is high in both lines [23, 37].

Studies have consistently shown injury-induced potentiation of glutamatergic synaptic transmission in the PBN→CeA pathway in multiple rodent models of persistent pain [4, 20, 21, 26, 27]. Our ex-vivo slice electrophysiology experiments further showed that intrinsic membrane and firing properties are unaltered in CeA-projecting PBN neurons following nerve injury (Fig. 2F–L), suggesting that plasticity of intrinsic excitability in these cells does not contribute to injury-induced hypersensitivity. Whether this lack of effect in intrinsic excitability is model-specific remains to be determined.

### CeA-projecting PBN neurons are major contributors to injury-induced pain sensitization but not baseline nociception

In-vivo and ex-vivo electrophysiological studies have shown injury-induced sensitization and potentiation of synaptic transmission in the PBN→CeA pathway in several models of persistent pain [4, 20, 21, 26, 27]. Our findings that chemogenetic inhibition of CeA-projecting PBN neurons reversed injury-induced hypersensitivity (Fig. 3B–E) and that chemogenetic activation induced hypersensitivity in the absence of injury (Fig. 5) in both male and female mice provide a causal link for the function of this pathway in pain processing following peripheral inflammation or nerve injury.

The analgesic effects we see after chemogenetic inhibition of the PBN→CeA pathway are specific to injured states. Thus, chemogenetic inhibition of CeA-projecting PBN neurons had no effect on withdrawal responses in sham-treated mice (Fig. 3F–I) or in the paw contralateral to nerve injury (Fig. 3B–E). These results are consistent with previous studies in naïve rodents that report no measurable effects on reflexive withdrawal responses to somatosensory stimuli after manipulations of PBN neurons that project to the CeA and express CGRP, tachykinin receptor 1, or mu-opioid receptors [12, 31–33]. The lack of modulation of reflexive responses to somatosensory stimuli by the PBN→CeA pathway in naïve mice, coupled with the reported function of this pathway in the modulation of aversion, fear memory, and pain-related affective-motivational responses [12, 31, 32] led to the suggestion that CeA-projecting PBN neurons contribute to the affective-motivational but not reflexive-defensive somatosensory component of pain. Our results indicate that CeA-projecting PBN neurons are critical to reflexive-defensive somatosensory responses but only in the context of injury. These results underscore the importance of including uninjured and injured states in studies investigating pain-related mechanisms.

Our formalin experiments further show that inhibition of the PBN→CeA pathway does not affect the first phase of the formalin test, which reflects direct activation of peripheral nociceptors [59], but selectively decreases the second phase, known to be mediated by peripheral and central sensitization [43, 45]. These results support the proposed function of the PBN→CeA pathway in injury-induced persistent pain, but not acute nociception. Our findings have potentially important clinical implications, providing insights towards the development of chronic pain treatment options that selectively target injured states.

### Modality-specific effects following activation of the PBN→CeA pathway

We show that chemogenetic inhibition of the PBN→CeA pathway reversed injury-induced hypersensitivity to tactile, deep tissue pressure, cold, and heat stimuli (Fig. 3B–E). In contrast, chemogenetic activation of this pathway induced hypersensitivity to tactile, deep tissue pressure, and cold stimulation (Fig. 5B–D) but not heat stimulation (Fig. 5E). These combined results suggest that spinal nociceptive inputs to the PBN are required for modulation of heat hypersensitivity by the PBN→CeA pathway but are not needed for modulation of tactile, cold and deep tissue pressure hypersensitivity.

Pain modality-specific effects are often observed in studies that manipulate pain circuits [22, 23, 47, 60, 61]. In addition, lamina I spinoparabrachial neurons have been shown to display modality selective responses in an ex-vivo semi-intact somatosensory preparation, with only a subpopulation of these neurons responding to peripheral heat stimulation [62]. Modality-specific function might be due to anatomical differences in the spinal inputs conveying heat vs tactile, cold and deep tissue pressure signals to the PBN. A possible scenario is that CeA-projecting PBN neurons receive direct inputs from spinal neurons that respond to tactile, cold and deep tissue pressure but not from spinal neurons that respond to heat stimuli. Experimental activation of the CeA-projecting PBN neurons would, therefore, mimic the activation normally driven by spinal inputs in response to cold, tactile and deep tissue pressure stimulation. In contrast, experimental activation of this pathway would not be sufficient to mimic the activation normally driven by spinal inputs in response to heat stimulation. Together, these findings support the idea that distinct circuits underlie modulation of specific sensory modalities.

### The PBN→CeA pathway contributes to both, stimulus-evoked reflexive-defensive responses and affective-motivational spontaneous responses to painful stimuli

Several pain-related responses to cutaneous noxious stimulation have been described [55–57, 63]. These behavioral responses have been further categorized as reflexive-defensive reactions (paw withdrawal) or affective-motivational responses (licking, biting, extended lifting, or guarding of the stimulated paw and escape responses such as jumping, hyperlocomotion or rearing) [55, 57, 59, 64]. A notable discovery from these prior studies is that distinct anatomical circuits modulate reflexive-defensive reactions vs affective-motivational responses to peripheral noxious stimuli [55, 63]. Our findings show that inhibition of the PBN→CeA pathway affects licking responses to formalin (Fig. 4D) suggesting this pathway modulates affective-motivational spontaneous responses to peripheral inflammation. These findings, combined with our results that chemogenetic manipulations of CeA-projecting PBN neurons also affects stimulus-evoked reflexive paw withdrawal responses after inflammation or nerve injury (Fig. 4E), suggest that the PBN→CeA pathway contributes to both reflexive-defensive reactions (stimulus-evoked paw withdrawal) and affective-motivational responses (spontaneous licking) in the context of injury.

We have shown that the CeA functions as a pain rheostat that bidirectionally modulates pain responses in a cell-type specific manner, with decreases driven by CeA-Som neurons and increases by CeA-PKCδ neurons [23]. In the present study we show that the PBN non-selectively targets both CeA-Som and CeA-PKCδ neurons. Whether subpopulations of PBN neurons send collaterals to both CeA-Som or CeA-PKCδ neurons or selectively target specific cell types in the CeA is unknown. Selective outputs from the PBN to CeA-Som or CeA-PKCδ neurons may have divergent functions in modulating pain-related behaviors as previously described for anatomically distinct outputs from the PBN to distinct brain regions [12]. This functional divergence might include projection-specific modulation of distinct pain modalities (i.e., heat, cold, deep tissue pressure, tactile), affective-motivational vs. reflexive-defensive responses and/or stimulus-evoked vs. spontaneous pain.

### Protective and maladaptive functions of the PBN→CeA in response to injury

The PBN has been described as a general alarm system to potential threats that orchestrates behavioral and physiological responses essential for survival [65]. Consistent with this notion, studies have shown PBN neurons are activated by several danger signals and this activation contributes to behavioral responses to threat in a cell-type and circuit-specific manner [14, 31, 66–74]. PBN neurons that express CGRP and project to the CeA, for example, have been shown to modulate escape responses to noxious heat and to contribute to fear memory [31]. Separate studies have also shown that manipulations of CeA-projecting PBN neurons modulate aversion and affective- motivational aspects of pain [12, 31, 32, 75, 76].

Maladaptive plasticity in the PBN→CeA pathway has also been proposed to contribute to persistent hypersensitivity in pathological conditions [18, 26], no longer serving a protective function. Consistent with this idea, recent work show that CGRP signaling in the right PBN→CeA pathway contributes to bladder pain-like behaviors but not pain-related aversion in a mouse model of cystitis [77]. Our results expand on these previous findings to show that the PBN→CeA pathway is also a major contributor of hypersensitivity to peripheral stimulation in response to inflammation or nerve injury. Based on these combined findings, we propose that this circuit functions as a biological alarm system that protects against further injury and promotes healing under physiological conditions. In pathological conditions, however, maladaptive changes in the PBN→CeA pathway might contribute to persistent hypersensitivity that is no longer protective.

### Supplementary information

Supplemental Material

Statistical table

Raw Data

## References

[1] Yong RJ, Mullins PM, Bhattacharyya N (2022). Prevalence of chronic pain among adults in the United States. Pain..

[2] Zimmer Z, Fraser K, Grol-Prokopczyk H, Zajacova A (2022). A global study of pain prevalence across 52 countries: examining the role of country-level contextual factors. Pain..

[3] Manion J, Waller MA, Clark T, Massingham JN, Neely GG (2019). Developing modern pain therapies. Front Neurosci.

[4] Sugimura YK, Takahashi Y, Watabe AM, Kato F (2016). Synaptic and network consequences of monosynaptic nociceptive inputs of parabrachial nucleus origin in the central amygdala. J Neurophysiol.

[5] Ma W, Peschanski M (1988). Spinal and trigeminal projections to the parabrachial nucleus in the rat: electron-microscopic evidence of a spino-ponto-amygdalian somatosensory pathway. Somatosens Res.

[6] Bernard JF, Peschanski M, Besson JM (1989). A possible spino (trigemino)-ponto-amygdaloid pathway for pain. Neurosci Lett.

[7] Jasmin L, Burkey AR, Card JP, Basbaum AI (1997). Transneuronal labeling of a nociceptive pathway, the spino-(trigemino-)parabrachio-amygdaloid, in the rat. J Neurosci.

[8] Bernard JF, Besson JM (1990). The spino(trigemino)pontoamygdaloid pathway: electrophysiological evidence for an involvement in pain processes. J Neurophysiol.

[9] Polgár E, Wright LL, Todd AJ (2010). A quantitative study of brainstem projections from lamina I neurons in the cervical and lumbar enlargement of the rat. Brain Res.

[10] Browne TJ, Hughes DI, Dayas CV, Callister RJ, Graham BA (2020). Projection neuron axon collaterals in the dorsal horn: placing a new player in spinal cord pain processing. Front Physiol.

[11] Browne TJ, Smith KM, Gradwell MA, Iredale JA, Dayas CV, Callister RJ (2021). Spinoparabrachial projection neurons form distinct classes in the mouse dorsal horn. Pain..

[12] Chiang MC, Nguyen EK, Canto-Bustos M, Papale AE, Oswald AM, Ross SE (2020). Divergent neural pathways emanating from the lateral parabrachial nucleus mediate distinct components of the pain response. Neuron..

[13] Hermanson O, Blomqvist A (1997). Subnuclear localization of FOS-like immunoreactivity in the parabrachial nucleus after orofacial nociceptive stimulation of the awake rat. J Comp Neurol.

[14] Chen JY, Campos CA, Jarvie BC, Palmiter RD (2018). Parabrachial CGRP neurons establish and sustain aversive taste memories. Neuron..

[15] Pauli JL, Chen JY, Basiri ML, Park S, Carter ME, Sanz E, et al. Molecular and anatomical characterization of parabrachial neurons and their axonal projections. Elife. 2022;11:e81868. 10.7554/eLife.81868 PMC9668336 36317965

[16] Chen Q, Heinricher MM (2019). Plasticity in the link between pain-transmitting and pain-modulating systems in acute and persistent inflammation. J Neurosci.

[17] Raver C, Uddin O, Ji Y, Li Y, Cramer N, Jenne C (2020). An amygdalo-parabrachial pathway regulates pain perception and chronic pain. J Neurosci.

[18] Neugebauer V, Li W, Bird GC, Han JS (2004). The amygdala and persistent pain. Neuroscientist..

[19] Bernard JF, Huang GF, Besson JM (1992). Nucleus centralis of the amygdala and the globus pallidus ventralis: electrophysiological evidence for an involvement in pain processes. J Neurophysiol.

[20] Neugebauer V, Li W (2003). Differential sensitization of amygdala neurons to afferent inputs in a model of arthritic pain. J Neurophysiol.

[21] Neugebauer V, Li W (2002). Processing of nociceptive mechanical and thermal information in central amygdala neurons with knee-joint input. J Neurophysiol.

[22] Carrasquillo Y, Gereau IVRW (2007). Activation of the extracellular signal-regulated kinase in the amygdala modulates pain perception. J Neurosci.

[23] Wilson TD, Valdivia S, Khan A, Ahn HS, Adke AP, Martinez Gonzalez S (2019). Dual and opposing functions of the central amygdala in the modulation of pain. Cell Rep.

[24] Veinante P, Yalcin I, Barrot M (2013). The amygdala between sensation and affect: a role in pain. J Mol Psychiatry.

[25] Hua T, Chen B, Lu D, Sakurai K, Zhao S, Han BX (2020). General anesthetics activate a potent central pain-suppression circuit in the amygdala. Nat Neurosci.

[26] Neugebauer V, Mazzitelli M, Cragg B, Ji G, Navratilova E, Porreca F (2020). Amygdala, neuropeptides, and chronic pain-related affective behaviors. Neuropharmacology..

[27] Li JN, Sheets PL (2020). Spared nerve injury differentially alters parabrachial monosynaptic excitatory inputs to molecularly specific neurons in distinct subregions of the central amygdala. Pain..

[28] Ji G, Li Z, Neugebauer V (2015). Reactive oxygen species mediate visceral pain-related amygdala plasticity and behaviors. Pain.

[29] Miyazawa Y, Takahashi Y, Watabe AM, Kato F (2018). Predominant synaptic potentiation and activation in the right central amygdala are independent of bilateral parabrachial activation in the hemilateral trigeminal inflammatory pain model of rats. Mol Pain.

[30] Thompson JM, Neugebauer V (2017). Amygdala plasticity and pain. Pain Res Manag.

[31] Han S, Soleiman MT, Soden ME, Zweifel LS, Palmiter RD (2015). Elucidating an affective pain circuit that creates a threat memory. Cell..

[32] Liu S, Ye M, Pao GM, Song SM, Jhang J, Jiang H (2022). Divergent brainstem opioidergic pathways that coordinate breathing with pain and emotions. Neuron.

[33] Barik A, Thompson JH, Seltzer M, Ghitani N, Chesler AT (2018). A brainstem-spinal circuit controlling nocifensive behavior. Neuron.

[34] Deng J, Zhou H, Lin JK, Shen ZX, Chen WZ, Wang LH (2020). The parabrachial nucleus directly channels spinal nociceptive signals to the intralaminar thalamic nuclei, but not the amygdala. Neuron..

[35] Feil K, Herbert H (1995). Topographic organization of spinal and trigeminal somatosensory pathways to the rat parabrachial and Kölliker-Fuse nuclei. J Comp Neurol.

[36] Saper CB (1995). The spinoparabrachial pathway: shedding new light on an old path. J Comp Neurol.

[37] Taniguchi H, He M, Wu P, Kim S, Paik R, Sugino K (2011). A resource of Cre driver lines for genetic targeting of GABAergic neurons in cerebral cortex. Neuron..

[38] Yalcin I, Megat S, Barthas F, Waltisperger E, Kremer M, Salvat E, et al. The sciatic nerve cuffing model of neuropathic pain in mice. J Vis Exp. 2014;51608. 10.3791/51608 PMC4217571 25078668

[39] Benbouzid M, Pallage V, Rajalu M, Waltisperger E, Doridot S, Poisbeau P (2008). Sciatic nerve cuffing in mice: a model of sustained neuropathic pain. Eur J Pain.

[40] Colburn RW, Lubin ML, Stone DJ, Wang Y, Lawrence D, D’Andrea MR (2007). Attenuated cold sensitivity in TRPM8 null mice. Neuron..

[41] Hargreaves K, Dubner R, Brown F, Flores C, Joris J (1988). A new and sensitive method for measuring thermal nociception in cutaneous hyperalgesia. Pain..

[42] Randall LO, Selitto JJ (1957). A method for measurement of analgesic activity on inflamed tissue. Arch Int Pharmacodyn Ther.

[43] Dubuisson D, Dennis SG (1977). The formalin test: a quantitative study of the analgesic effects of morphine, meperidine, and brain stem stimulation in rats and cats. Pain..

[44] Tjolsen A, Berge OG, Hunskaar S, Rosland JH, Hole K (1992). The formalin test: an evaluation of the method. Pain..

[45] Abbott FV, Franklin KBJ, Westbrook FR (1995). The formalin test: scoring properties of the first and second phases of the pain response in rats. Pain..

[46] Adke AP, Khan A, Ahn HS, Becker JJ, Wilson TD, Valdivia S, et al. Cell-type specificity of neuronal excitability and morphology in the central amygdala. eNeuro. 2021;8. 10.1523/ENEURO.0402-20.2020 PMC7877473 33188006

[47] Singh S, Wilson TD, Valdivia S, Benowitz B, Chaudhry S, Ma J, et al. An inhibitory circuit from central amygdala to zona incerta drives pain-related behaviors in mice. Elife. 2022;11:e68760. 10.7554/eLife.68760 PMC9635874 36269044

[48] Amano T, Amir A, Goswami S, Paré D (2012). Morphology, PKCδ expression, and synaptic responsiveness of different types of rat central lateral amygdala neurons. J Neurophysiol.

[49] Haubensak W, Kunwar PS, Cai H, Ciocchi S, Wall NR, Ponnusamy R (2010). Genetic dissection of an amygdala microcircuit that gates conditioned fear. Nature..

[50] Zajdel J, Skold J, Jaarola M, Singh AK, Engblom D (2021). Calcitonin gene related peptide alpha is dispensable for many danger-related motivational responses. Sci Rep.

[51] Luo T, Yu S, Cai S, Zhang Y, Jiao Y, Yu T (2018). Parabrachial neurons promote behavior and electroencephalographic arousal from general anesthesia. Front Mol Neurosci.

[52] Campos CA, Bowen AJ, Roman CW, Palmiter RD (2018). Encoding of danger by parabrachial CGRP neurons. Nature..

[53] Choi S, Hachisuka J, Brett MA, Magee AR, Omori Y, Iqbal NU (2020). Parallel ascending spinal pathways for affective touch and pain. Nature..

[54] Backonja MM, Stacey B (2004). Neuropathic pain symptoms relative to overall pain rating. J Pain.

[55] Huang T, Lin SH, Malewicz NM, Zhang Y, Zhang Y, Goulding M (2019). Identifying the pathways required for coping behaviours associated with sustained pain. Nature..

[56] Corder G, Doolen S, Donahue RR, Winter MK, Jutras BL, He Y (2013). Constitutive μ-opioid receptor activity leads to long-term endogenous analgesia and dependence. Science..

[57] Corder G, Tawfik VL, Wang D, Sypek EI, Low SA, Dickinson JR (2017). Loss of μ opioid receptor signaling in nociceptors, but not microglia, abrogates morphine tolerance without disrupting analgesia. Nat Med.

[58] Allen HN, Bobnar HJ, Kolber BJ (2021). Left and right hemispheric lateralization of the amygdala in pain. Prog Neurobiol.

[59] Yi M, Zhang H, Lao L, Xing GG, Wan Y (2011). Anterior cingulate cortex is crucial for contra- but not ipsi-lateral electro-acupuncture in the formalin-induced inflammatory pain model of rats. Mol Pain.

[60] Yang H, de Jong JW, Cerniauskas I, Peck JR, Lim BK, Gong H (2021). Pain modulates dopamine neurons via a spinal-parabrachial-mesencephalic circuit. Nat Neurosci.

[61] Samineni VK, Grajales-Reyes JG, Copits BA, O’Brien DE, Trigg SL, Gomez AM, et al. Divergent modulation of nociception by glutamatergic and GABAergic neuronal subpopulations in the periaqueductal gray. eNeuro. 2017;4. 10.1523/ENEURO.0129-16.2017 PMC5370278 28374016

[62] Hachisuka J, Koerber HR, Ross SE (2020). Selective-cold output through a distinct subset of lamina I spinoparabrachial neurons. Pain..

[63] Corder G, Ahanonu B, Grewe BF, Wang D, Schnitzer MJ, Scherrer G (2019). An amygdalar neural ensemble that encodes the unpleasantness of pain. Science..

[64] Bolles RC, Fanselow MS (1980). A perceptual-defensive-recuperative model of fear and pain. Behav Brain Sci.

[65] Palmiter RD (2018). The parabrachial nucleus: CGRP neurons function as a general alarm. Trends Neurosci.

[66] Mumphrey MB, Hao Z, Townsend RL, Patterson LM, Münzberg H, Morrison CD (2016). Eating in mice with gastric bypass surgery causes exaggerated activation of brainstem anorexia circuit. Int J Obes.

[67] Sato M, Ito M, Nagase M, Sugimura YK, Takahashi Y, Watabe AM (2015). The lateral parabrachial nucleus is actively involved in the acquisition of fear memory in mice. Mol Brain.

[68] Uddin O, Studlack P, Akintola T, Raver C, Castro A, Masri R (2018). Amplified parabrachial nucleus activity in a rat model of trigeminal neuropathic pain. Neurobiol Pain.

[69] Kaur S, Wang JL, Ferrari L, Thankachan S, Kroeger D, Venner A (2017). A genetically defined circuit for arousal from sleep during hypercapnia. Neuron..

[70] Rodriguez E, Sakurai K, Xu J, Chen Y, Toda K, Zhao S (2017). A craniofacial-specific monosynaptic circuit enables heightened affective pain. Nat Neurosci.

[71] Li J, Ali MSS, Lemon CH (2022). TRPV1-lineage somatosensory fibers communicate with taste neurons in the mouse parabrachial nucleus. J Neurosci.

[72] Crawford LS, Mills EP, Hanson T, Macey PM, Glarin R, Macefield VG (2021). Brainstem mechanisms of pain modulation: a within-subjects 7T fMRI study of placebo analgesic and nocebo hyperalgesic responses. J Neurosci.

[73] Roeder Z, Chen Q, Davis S, Carlson JD, Tupone D, Heinricher MM (2016). Parabrachial complex links pain transmission to descending pain modulation. Pain..

[74] Li L, Ding J, Ren Z, Han Q, Hu G, Xiao M (2006). Expression and colocalization of NADPH-diaphorase and Fos in the subnuclei of the parabrachial nucleus in rats following visceral noxious stimulation. Brain Res.

[75] Kang SJ, Liu S, Ye M, Kim DI, Pao GM, Copits BA (2022). A central alarm system that gates multi-sensory innate threat cues to the amygdala. Cell Rep.

[76] Ito M, Nagase M, Tohyama S, Mikami K, Kato F, Watabe AM (2021). The parabrachial-to-amygdala pathway provides aversive information to induce avoidance behavior in mice. Mol Brain.

[77] Allen HN, Chaudhry S, Hong VM, Lewter LA, Sinha GP, Carrasquillo Y (2023). A parabrachial-to-amygdala circuit that determines hemispheric lateralization of somatosensory processing. Biol Psychiatry.

