# Supplementary material for: The parabrachial to central amygdala pathway is critical to injury-induced pain sensitization in mice
Source: Neuropsychopharmacology. 2023 Aug 4;49(3):508–20. doi: 10.1038/s41386-023-01673-6 (PMC10789863; doi:10.1038/s41386-023-01673-6)
Supplement: Supplementary file 1 — Supplemental Material [file 41386_2023_1673_MOESM1_ESM.pdf]

536 ## Supplementary figures

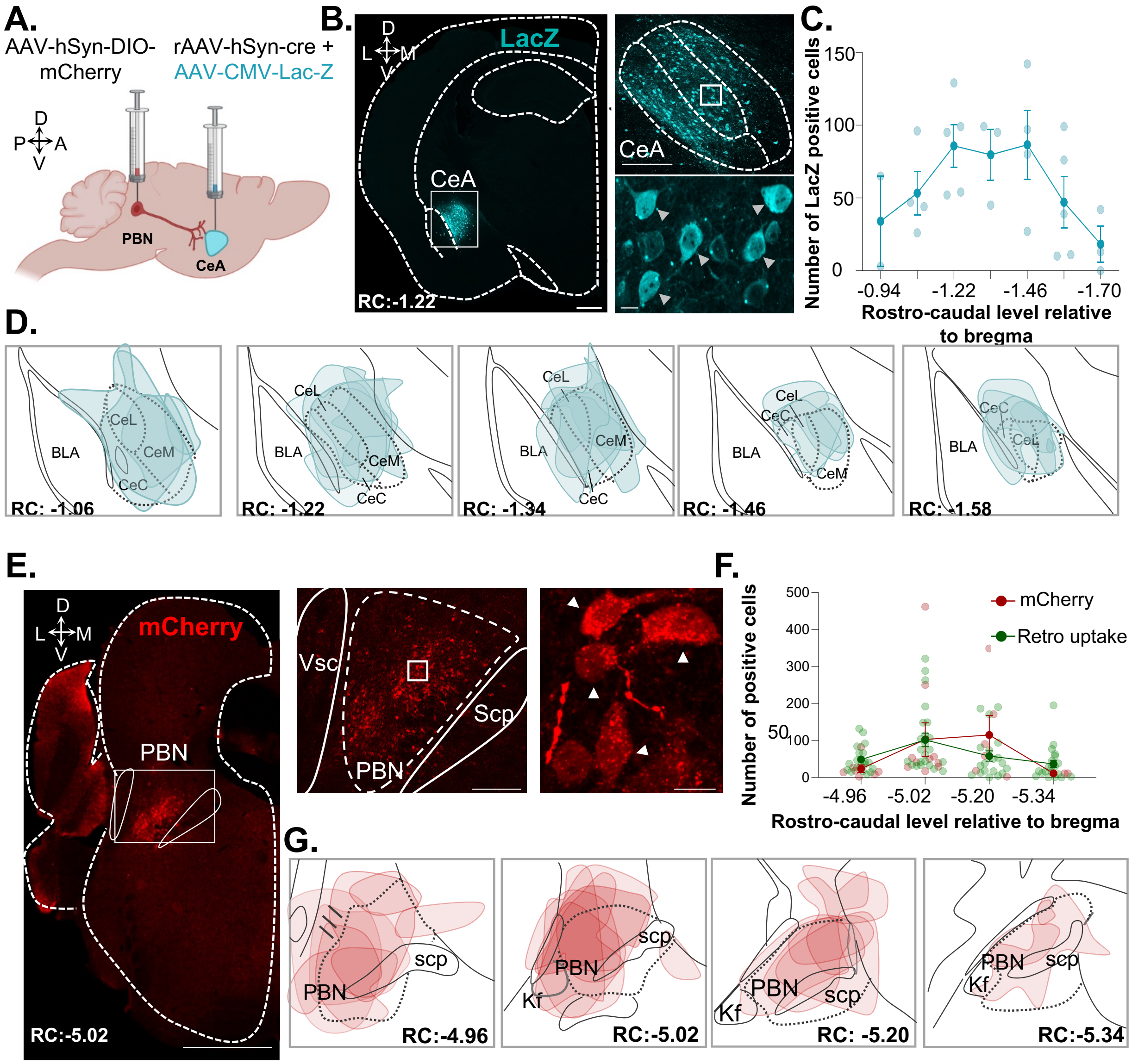

537 ## Figure S1

Supplementary:

Figure S1: Validation of intersectional genetic approach. (A) Schematic of intersectional approach. Male C57BL/6J mice were stereotaxically co-injected with AAV8-hSyn-DIO-mcherry into the right PBN and with a mix (1:1) of AAV.hSyn.HI.eGFP-Cre and pAAV.CMV.LacZ.bGH into the right CeA. **(B)** Representative images of the pAAV.CMV.LacZ.bGH injection into the CeA in a coronal brain slice. Immunofluorescence for LacZ is shown in cyan. Left panel shows low magnification image. Scale bar represents 500 μm. Right panel shows high magnification images of area delineated by the white box. Scale bar for the top panel represents 200 μm and 10 μm for the bottom. Arrows represent transduced cells. Rostro-caudal level relative to bregma is -1.22. **(C)** Number of LacZ transduced cells by rostro-caudal distribution (n=4 mice and four to six slices per mouse). **(D)** Drawings showing the rostro-caudal distribution of the pAAV.CMV.LacZ.bGH injection into the CeA. **(E)** Representative images of the AAV8-hSyn-DIO-mcherry injection into the PBN in a coronal brain slice. Immunofluorescence for mCherry is shown in red. Left panel shows low magnification image. Scale bar represents 500 μm. Middle and left panels show high magnification images of area delineated by white box. Arrows represent transduced cells. Scale bar for the middle panel represents 200 μm and 10 μm for the left. RC is -4.96. **(F)** Number of cells with retrograde uptake following AAV.hSyn.HI.eGFP-Cre injection and mCherry-transduced cells by RC. n=10 mice for mCherry and n=25 mice for retrograde uptake; 2-4 slices per mouse. All data is presented as means ± SEM. **(G)** Drawings showing the rostro-caudal distribution of the AAV8-hSyn-DIO injection into the PBN. Abbreviations: superior cerebellar peduncle (scp), parabrachial nucleus (PBN), central amygdala (CeA), central

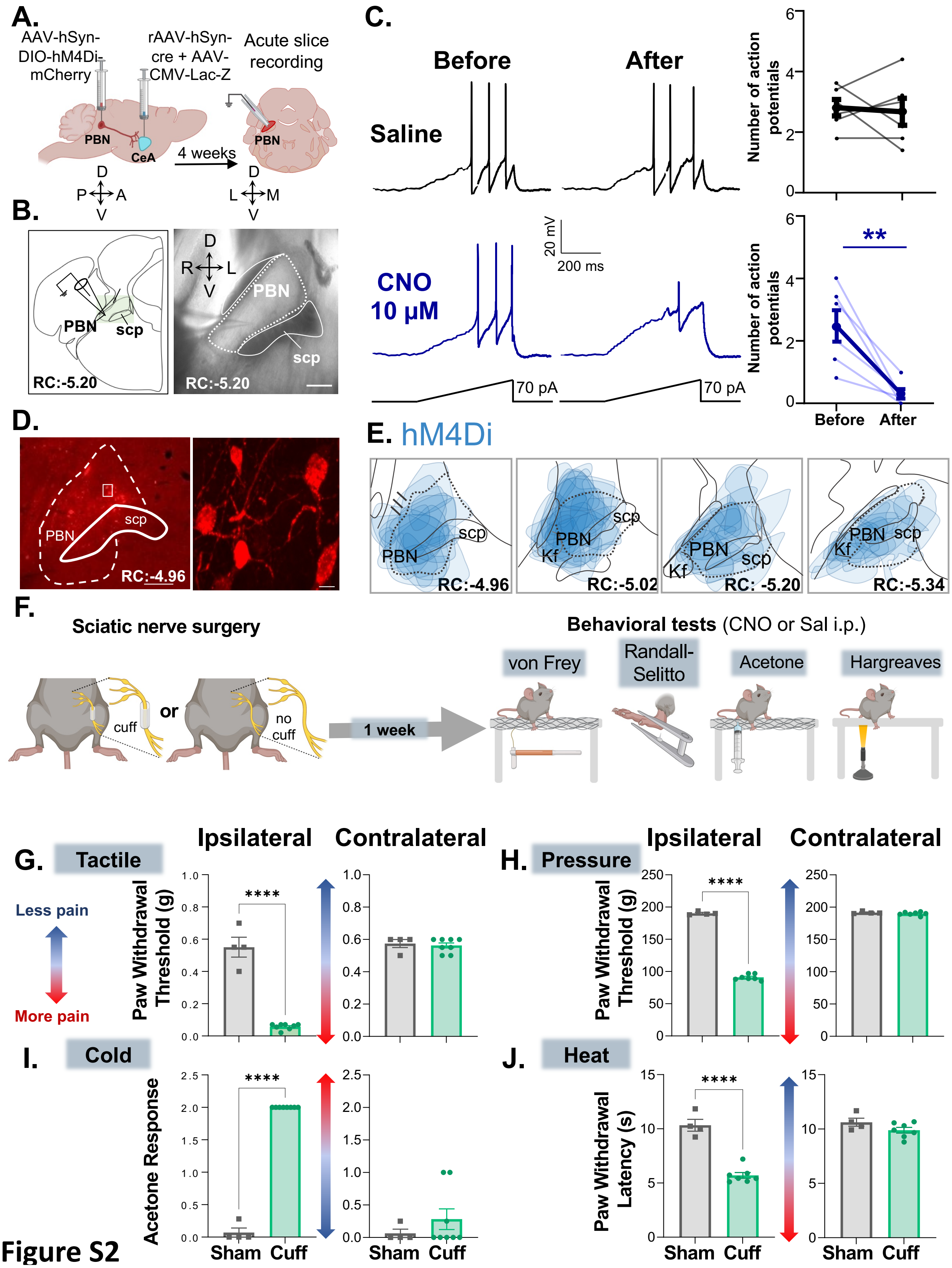

538 **Figure S2**

539

amygdala medial (CeM), central amygdala lateral (CeL), central amygdala capsular (CeC), basolateral amygdaloid (BLA).

Figure S2: Validation of chemogenetic intersectional approach and behavioral assays. (A) Timeline of acute slice electrophysiology experiments. Male C57BL/6J mice were stereotaxically injected with AAV8-hSyn-DIO-hMD4i into the PBN and AAV.hSyn.HI.eGFP-Cre into the CeA. Acute slice recordings were obtained from hMD4i-transduced PBN neurons 4 weeks after the injection. **(B)** Left panel: schematic description (left) and representative differential interference contrast image (right) of coronal brain slice with recording electrode in PBN. Scale bar represents 150 μm. RC: -5.20. **(C)** Representative voltage traces of recordings obtained from hMD4i-transduced PBN neurons in response to depolarizing current injections before (left) and after (right) bath application of saline (top) or 10 μM CNO (bottom). Number of action potentials before and after bath applications. n=6 cells per treatment. Paired two tailed t test; $^{**}p<0.01$. **(D)** Representative image of the AAV8-hSyn-DIO-hMD4i injection into the PBN in a coronal brain slice. Immunofluorescence for mCherry is shown in red. Left panel shows a low magnification image and right panel a high magnification of RC: -4.96. Scale bar for left panel is 125 μM and 10 μM for right panel. **(E)** Drawings showing the rostro-caudal distribution of AAV8-hSyn-DIO-hMD4i injection into the PBN. **(F)** Experimental timeline of behavioral experiments. Sciatic nerve cuff or sham surgery was performed in male C57BL/6J mice. Following 1 week of recovery, von Frey, Randall-Selitto, Acetone and Hargreaves tests were used to address sensitivity to tactile, pressure, cold and heat stimulation, respectively, in the hind paws ipsilateral and contralateral to cuff treatment.

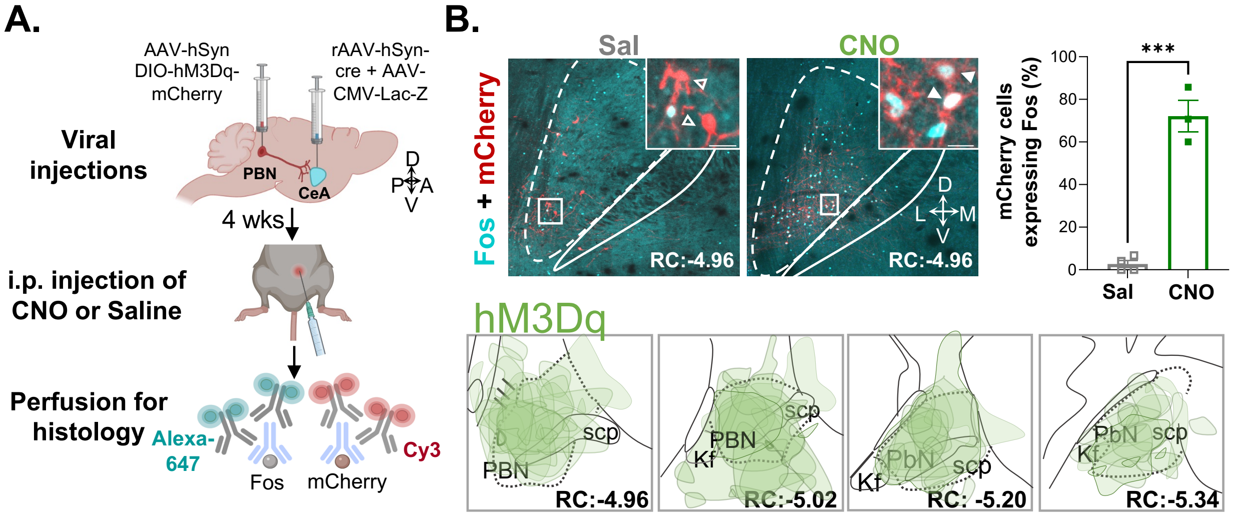

540 **Figure S3**

(G-J) Paw withdrawal threshold after tactile **(G)** pinch **(H)** stimulation, acetone response score **(I)** and withdrawal latency after heat stimulation **(J)** of the hind paw ipsilateral or contralateral to sham and cuff treatments. n=4 mice for sham and n=8 mice for cuff in all tests. Unpaired two tailed t test; ****$p<0.0001$. Individual mice are represented by scatter points. Abbreviations: ventral spinocerebellar tract (vsc), superior cerebellar peduncle (scp), Kölliker-Fuse nucleus (kf). All data is presented as means ± SEM.

Figure S3: Validation of CNO-mediated activation of CeA-projecting PBN neurons

(A) Timeline of chemogenetic activation validation. Male C57BL/6J mice were stereotaxically injected with AAV8-hSyn-DIO-hMD3q into the right PBN and a mix (1:1) of AAV.hSyn.HI.eGFP-Cre and pAAV.CMV.LacZ.bGH into the right CeA. 4 weeks after, mice were intraperitoneally injected with either CNO or saline and perfused 30-45mins after for immunohistology purposes. **(B)** Representative images of PBN slices of stereotaxically injected mice after saline (left) and CNO (right) i.p. injections. Immunofluorescence for mCherry (representing hM3Dq-transduced cells) is shown in red and for Fos in cyan color. Insets: solid arrows represent mCherry+ cells colocalized with Fos while open arrows represent mCherry+ only cells. Scale bar represents 150 μm for low magnification and 10 μm for insets. RC for both pictures: -4.96. Right panel shows mean ± SEM percentage of mCherry+ cells expressing c-Fos. n=4 mice for saline, n=3 mice for CNO; 4 slices per mouse. Unpaired two tailed t test; ***$p<0.001$. Bottom panel shows drawings of the rostro-caudal distribution of the AAV8-hSyn-DIO-hMD3q injection into the PBN.

Materials and Methods

Subjects

Experiments were approved by the Animal Care and Use Committee of the National Institute of Neurological Disorders and Stroke and the National Institute on Deafness and other Communication Disorders with the guidance from the National Institutes of Health (NIH). Adult C57BL/6J or Swiss Webster mice between the age of 8 to 17 weeks old, bred in house or purchased from Jackson Laboratory, were used for all behavioral and histological experiments. The sex of the mice used for each experiment is described in the sections below. The following mouse lines were used for electrophysiology experiments: heterozygous *Prkcd*-cre mice (GENSAT – founder line 011559-UCD) or heterozygous male *Sst*-cre (Jackson Laboratory – founder line 018973) crossed with homozygous Ai9 mice (Jackson Laboratories – founder line 007909). Offspring mice were genotyped for cre-recombinase using tail biopsies and PCR (Transnetyx) with the following primers: TTAATCCATATTGGCAGAACGAAAACG (forward) and CAGGCTAAGTGCCTTCTCTACA (reverse). The expression and fidelity of Cre in Som+ and PKCδ+ neurons have been previously described [1,2]. Mice were initially group housed (up to 5 mice per cage) with littermates of the same sex in a vivarium with controlled humidity and temperature under reversed 12 h light/dark cycle (9 pm to 9 am light). Following surgery, pairs of littermate mice of the same sex and pain treatment were transferred to new home cages with perforated Plexiglass dividers to keep one mouse per side. All behavioral tests were performed under red light during the dark period, between the hrs of 10 am and 6 pm. Mice received one handling session per day for at least 5 days before the start of behavioral and electrophysiological experiments as

previously described [3]. During each handling session, mice were allowed to move freely on the hands of the experimenter for approximately 5-8 min and were then injected with 50-100 µl saline intraperitoneally (i.p.). On the first surgery day, animals from the same litter were randomly assigned to experimental groups (i.e., brain injection and sciatic nerve treatments). All subsequent experiments and analyses were performed blind to experimental treatment.

Stereotaxic injections

Acute microinjections were performed using a small animal stereotaxic instrument (David Kopf Instruments). Male and female C57BL/6J and Swiss Webster mice were initially anesthetized with 5% isoflurane in preparation for the stereotaxic surgery. After induction, mice were maintained with 2% isoflurane at a flow rate of 0.5 L/min for the duration of surgery. A hand warmer (Hot Rods Hand Warmers) was used for thermal maintenance during the procedure. Stereotaxic injections were performed using 0.5 µl Hamilton Neuros 32-gauge syringes (Neuro model 7000.5 KH) at a flow rate of 0.1 µl/min. The syringe was left in place for an additional 15 min to allow for diffusion of virus and to prevent backflow.

Based on previous literature demonstrating hemispheric lateralization of CeA function in the modulation of hypersensitivity, [4-7] stereotaxic injections were performed in the right hemisphere in all experiments. For intersectional chemogenetic experiments, the CeA of C57BL/6J or Swiss Webster mice was injected with either 0.05 µl of pENN.AAV.hSyn.HI.eGFP-Cre.WPRE.SV40 (Addgene 105540-AAVrg) or 0.07 µl of a 1:1 mixture of pENN.AAV.hSyn.HI.eGFP-Cre.WPRE.SV40 (Addgene 105540-AAVrg) and pAAV.CMV.LacZ.bGH (Addgene 105531-AAV8). During the same surgery, the PBN was

injected with 0.1 µl of AAV8-hSyn-DIO-hM4D(Gi)-mCherry (Addgene 44362-AAV8) or AAV8-hSyn-DIO-mcherry (Addgene 50459-AAV8) or AAV8-hSyn-DIO-hM3D(Gq)-mCherry (Addgene 44361-AAV8). The position of specific brain regions relative to bregma, midline and surface of the skull has been shown to be strain specific [8]. For this reason, optimal coordinates for specific brain regions per strain were determined by injecting Evans Blue dye. Correct targeting was assessed based on dye location using distinctive anatomical landmarks and a mouse brain atlas for both the CeA and the PBN.

The following stereotaxic coordinates were used to target the CeA in C57BL/6J mice: 1.25 mm posterior from bregma, 2.95 mm lateral to midline, 4.5 mm ventral to skull surface. PBN injections were performed using the following stereotaxic coordinates: 4.9 mm posterior from bregma, 1.2 mm lateral to midline, 3.78 mm ventral to skull surface. To target the CeA in Swiss Webster mice, the following coordinates were used: 1.4 mm posterior from bregma, 3.2 mm lateral to midline and 4.8 mm ventral to skull surface. For PBN injections the following coordinates were used: 5.0 mm posterior to bregma, 1.3 lateral to midline and 3.52 mm ventral to skull surface. Mice recovered for 3 weeks before additional experimental procedures. At the end of the experiments, mice were transcardially perfused with 4% paraformaldehyde solution in 0.1 M Phosphate Buffer (PFA/PB), pH 7.4. Injection sites were verified through histology and only animals with correct injection sites were included in the analyses.

For CeA slice electrophysiology experiments, 0.1 µl of rAAV1-hSyn-hChR2(H134R)-EYFP- (Addgene 26973) was injected into the right PBN of *Sst*-cre::Ai9 or *Prkcd*-cre::Ai9 mice. Injections were performed using the following stereotaxic coordinates for *Prkcd*-cre::Ai9: 5.2 mm posterior from bregma, 1.3 mm lateral to midline,

3.52 mm ventral to skull surface. For *Sst*-cre::Ai9 mice coordinates were: 5.1 mm posterior from bregma, 1.2 mm lateral to midline, 3.78 mm ventral to skull surface. Electrophysiology experiments were performed at least 4 weeks after viral injection to allow for expression of ChR2 in PBN terminals in the CeA.

For PBN slice electrophysiology experiments, 0.22 µl of red or green Retrobeads™ IX (Lumafluor Inc) were stereotaxically injected into the right CeA of male C57/BL6J mice using the following coordinates: 1.25 posterior from bregma, 2.95 lateral to midline, and -4.85 ventral to skull surface. These animals received concurrent sciatic cuff implantation in the right sciatic nerve, described in the following section.

Sciatic cuff implantation

Sciatic nerve surgeries were performed 3 weeks after stereotaxic surgeries as described [9,10]. Male and female C57BL/6J or male Swiss Webster mice from the same litter were randomly assigned to either cuff or sham surgeries such that pairs of co-housed mice underwent the same manipulation. Mice were anesthetized with 2% isoflurane at a flow rate of 0.5 L/min and an incision 1 cm long was made in the proximal one third of the left lateral thigh. The sciatic nerve was exposed and gently stretched with forceps inserted under the nerve. The cuff group was implanted with a 2-mm-long piece of PE-20 non-toxic sterile polyethylene tubing (0.38 mm ID / 1.09 mm OD; Daigger Sci) that was split along its side and slid onto the exposed sciatic nerve. After cuff implantation, the nerve was returned to the thigh. For sham animals, the sciatic nerve was exposed and gently stretched using forceps and then returned to its normal position. The skin was closed with wound clips (Reflex Clips, World Precision Instruments). Mice recovered for at least a

one-week before undergoing behavioral testing. Wound clips were not actively removed during the experiment to minimize stress, discomfort, and pain during behavior testing days (7-12 days after sciatic nerve surgery). All experiments were replicated at least twice.

Nociceptive testing

Male and female mice were used for nociceptive testing. Testing on males and females was performed separately. Behavioral experiments were not directly powered to detect sex differences, but no overt sex differences were observed. Thus, data from both sexes were pooled and individual data points for each sex are clearly identified in all scatter plots. Experimenter was blind to treatment for all behavioral testing and every cohort was counterbalanced to include mice from all experimental groups. Testing was performed on two consecutive days per test. On each testing day, baseline (pre-injection) measurements were taken. Saline or Clozapine-N-oxide (CNO, Enzo Life Sciences, Farmingdale, NY) was injected i.p. (10 mg/kg body weight for hM4Di mice or 3 mg/kg body weight for hM3Dq) and a second measurement (post-injection) was taken 30 minutes to 45 minutes after the i.p. injection. Mice were randomly assigned into control (saline) or experimental (CNO) group on the first day of each test. The next day, the tests were performed with the opposite treatment. The order of i.p. injections was counterbalanced in these experiments. The von Frey and acetone evaporative tests were performed on the same day, waiting 30 minutes between tests, and 7-8 days after the cuff implantation. Heat sensitivity test was performed 9-10 days after cuff implantation

and the Randall-Selitto test for pressure sensitivity on days 11-12. Testing boxes for all tests were 11 × 11 × 13 cm, ventilated and made of opaque Plexiglas (custom-made).

von Frey test

Mice were habituated (for 3 h) to testing chambers placed on an elevated mesh platform (custom-made) prior to behavioral assessment. von Frey filaments (North Coast Medical, Inc. San Jose, CA) were used to measure sensitivity to tactile stimulation as described [11]. Starting with the smallest filament, each von Frey filament, was applied to the mouse hind paws until bent at 30° for ~2 s. The smallest filament that elicited a paw withdrawal response in at least three of five trials was taken as the paw withdrawal threshold for that trial. The average of 3-5 measurements was calculated individually for each paw and used as the withdrawal threshold.

Acetone evaporative test

An adapted acetone evaporative test [12] was used to measure sensitivity to a cold stimulus. An acetone drop was formed at the top of a 1 ml or 3 ml syringe then lightly applied through the wire mesh to the plantar surface of the hind paw ipsilateral (treated) or contralateral (untreated) to sciatic nerve surgery. Following acetone application, nociceptive responses were scored based on responses observed for 60 s post application. A modified version of the scoring system described for this test [13] was used, with 0 = a rapid transient lifting, licking, or shaking of the hind paw, which subsides immediately; 1 = lifting, licking, and/or shaking of the hind paw, that continues beyond the initial application, but fades within 5 s; 2 = protracted, repeated lifting, licking, and/or shaking of the hind paw. The average score of 3-5 stimulations were taken from and used for each hind paw.

Hargreaves test

A modified version of the Hargreaves test [14] and the Plantar Analgesia Meter (IITC Life Sciences, Woodland Hills, CA) was used to measure heat sensitivity as described [15]. Animals were habituated for 1 hr to a Plexiglas testing chamber on an elevated platform with a clear glass surface heated to 30°C. The thermal stimulus was a constant radiant heat source with an active intensity of 25 for C57BL/6J or 32 for Swiss Webster mice directed to the hind paw plantar surface. Active intensity is the intensity of light source as defined by the manufacturer. The time each mouse needed to withdraw the hind paw was recorded. A 15-second cutoff was used to prevent injury. The average of five withdrawal latencies were taken from and used for each hind paw.

Randall-Selitto test

A modified Randall-Selitto test [16] and the Digital Paw Pressure Randall-Selitto meter (IITC Life Sciences, Woodland Hills, CA) was used to measure response thresholds to mechanical pressure stimuli in deep tissue. This is an assay developed and traditionally used in physically restrained rats as mice rarely tolerate such handling [17-21]. To avoid potential unwanted effects of restraining in our experiments, male or female mice were lightly anesthetized with 3% isoflurane in an induction chamber, then maintained with 0.5%–1% isoflurane at a flow rate of 0.5 L/min. A sharp pinch not exceeding 200 g of force was delivered to the plantar surface of the paw ipsilateral and contralateral to cuff or sham implanted sciatic nerve. Pinch pressure for withdrawal was recorded and the average of five trials per hind paw was calculated individually for each animal.

Formalin test

Male and female C57BL/6J or male Swiss Webster mice were habituated for 1 hr in plexiglass testing chambers on an elevated platform with transparent floors. A mirror was positioned directly below the chambers to properly visualize mice hind paws. After habituation, mice received i.p. injection of either CNO (10 mg/kg) or saline and were immediately returned to the testing chamber. The experimenter was blind to treatment. 30-40 minutes after i.p. injection, C57BL/6J mice were injected with 10 μl of 2-3% formalin and Swiss Webster with 10 μl of 5% formalin into either left or right hind paws. Immediately after, they were returned to the testing chambers and time spent in nociceptive behaviors, defined as licking, lifting, and shaking the hind paws, were individually measured for 40 min (C57BL/6J) or 60 min (Swiss Webster) in 5 min intervals. Total time spent in spontaneous nociceptive behaviors was defined as the sum of the time spent in the individual behaviors. Phase 1 of the formalin test was defined as the first five minutes post-formalin injection and phase 2 was measured from 5 to 40 min after formalin injection. Sensitivity to tactile stimulation was measured in male Swiss Webster mice one day after formalin injection using von Frey filaments as described above.

Immunohistochemistry

At the end of the experiments, mice were deeply anesthetized with 1.25% Avertin anesthesia (2,2,2-tribromoethanol and tert-amyl alcohol in 0.9% NaCl; 0.025 ml/g body weight), then perfused transcardially with 0.9% NaCl (37°C), followed by 100 mL of ice-cold 4% paraformaldehyde in phosphate buffer (PFA/PB). Immediately after perfusion, we dissected the brains, post fixed in 4% PFA/PB overnight at 4°C and cryoprotected in 30% sucrose/PB for 48 hr. Thirty μm coronal sections containing the regions of interest

(central amygdala and/or parabrachial nucleus) were collected in 0.1 M phosphate buffered saline (PBS), pH 7.4 containing 0.01% sodium azide (Sigma) using a freezing sliding microtome. Sections were stored in 0.1 M PBS, pH 7.4 containing 0.01% sodium azide (Sigma) at 4°C until used for immunostaining. After rinsing in PBS, sections were incubated in 0.1% Triton X-100 in PBS for 10 min at room temperature and were then blocked in 5% normal goat serum (NGS) (Vector Labs, Burlingame, CA) with 0.1% Triton X-100, 0.05% Tween-20 and 1% bovine serum albumin (BSA) for 30 min at room temperature. Primary antibody incubations were for overnight or 72 hr at 4°C, followed by 1 hr at room temperature. Sections were then rinsed in PBS and incubated in secondary antibodies in 1.5% NGS blocking solution with 0.1% Triton X-100, 0.05% Tween 20 and 1% BSA, protected from light, for 2 hr at room temperature. Sections were then rinsed in PBS, mounted on positively charged glass slides and air-dried overnight. Coverslips were placed using DAPI Fluoromount-G mounting media (Southern Biotech) and slides were stored at room temperature overnight and then stored under 4°C.

Verification of brain injection sites for behavioral experiments

For injection site verification, the following primary and secondary antibodies were used: rat anti-mCherry (1:250 for 72 hr or 1:125 overnight, Invitrogen, M11217), chicken anti-GFP (1:1000 for 72 hr or 1:500 for overnight, Invitrogen, ab13970), rabbit anti-β-gal (1:1000, Millipore Sigma, ab986 for 72 hr or 1:500 overnight) and goat anti-rat Cy3 (1:250, Invitrogen, A10522), Alexa Fluor 647-conjugated goat anti-rabbit (1:500, Invitrogen, A21244) and/or Alexa Fluor 488-conjugated goat anti-chicken IgY (H+L) (1:1000, Invitrogen, A-11039). Correct injection sites were defined as brains with mCherry+ cells

localized to the PBN and mCherry+ terminals and LacZ transduced cell bodies localized to the CeA.

Fos monitoring

For Fos experiments to validate CNO-mediated activation of neurons expressing hM3dq, mice were habituated for 1 hr in a behavioral room with red lights. Mice then received i.p. injection of either CNO (3 mg/kg) or saline and were immediately returned to their cages. 30-35 minutes after saline or CNO i.p. injections, animals were then anesthetized with 1.25% Avertin i.p. (0.4 mg/g) injection and perfused as previously described. For pinch-induced Fos experiments, pinch pressure for withdrawal was recorded at 1-minute intervals for 30 minutes in male and female mice. Mice were then euthanized and perfused as described above 1 hr after the completion of the Randall-Selitto test. For both Fos experiments, the following primary antibodies were used: rat anti-mCherry (1:250 for 72 hr or 1:125 overnight, Invitrogen, M11217) and rabbit anti-Phospho-Fos (Ser32) (1:2000 for 72 hr or 1:1000 overnight, Cell Signaling Technology, #5348). For secondary antibodies**:** goat anti-rat Cy3 (1:250, Invitrogen, A10522) and Alexa Fluor 647-conjugated goat anti-rabbit (1:500, Invitrogen, A21244) were used.

Immunohistochemistry to verify injection sites for electrophysiological experiments

Slices were fixed in 4% PFA at 4 °C for 48 hr at the end of recordings. Following slice fixation, slices containing the CeA and PBN were stored in 0.1% sodium azide in PBS 4 °C until histological processing. Slices were rinsed in 0.1 M PBS and incubated in 0.1% Triton-X-100 in PBS for 10 min at room temperature. 5% NGS-based blocking buffer

(0.1% Triton-X-100, 0.05% Tween-20, and 1% BSA) was used for slice incubation at room temperature for 30 minutes. Slices were then rinsed in PBS and incubated in 1.5% NGS-based blocking buffer (0.1% Trition-X-100, 0.05% Tween-20, and 1% BSA) containing the primary antibody rabbit anti-GFP (Invitrogen, A6455, 1:250 concentration) for 1 week at 4 °C followed by 1 hr at room temperature. Slices were then rinsed in PBS and incubated in 1.5% NGS-based blocking buffer (0.1% Trition-X-100, 0.05% Tween-20, 1% BSA) containing the secondary antibody goat anti-Rabbit IgG (H+L) Highly Cross-Adsorbed (Alexa Fluor 488, Invitrogen A11034, 1:100 concentration) overnight at room temperature under minimal light. Following secondary antibody incubation, slices were rinsed in PBS and incubated for 10 min in a series of increasing concentrations of 2,2'-thiodiethanol (TDE, Sigma) to allow for tissue clearing prior to image acquisition [22] in the following order of concentrations of TDE in PBS: 10%, 30%, 60%, and 80% followed by a 2-hr incubation in 97% TDE solution. All slice incubations in TDE were at room temperature. Slices were mounted on positively charged glass slides, coverslips were placed on slices covered with 97% TDE, and slides were sealed with either clear nail polish or Fluoromount-G mounting media (SouthernBiotech).

For optogenetic assisted circuit mapping, correct injection sites were defined as brains with localized GFP-expressing cell bodies at the PBN and GFP+ terminals at the CeA. For electrophysiological experiments to validate intersectional approach, correct injection sites were defined as brains with GFP-expressing transduced cells at the CeA, mCherry+ transduced cells at the PBN and mCherry-expressing terminals at the CeA.

Image acquisition and analysis

Images were acquired using a Nikon A1R laser scanning confocal microscope or a Leica DM5500 using a 2X (for low magnification) or a 10X (for high magnification) objective. 40X oil-immersion objective was used for high magnification representative images. Experimenter was blind to experimental group and analyses were performed on images collected using a 10X objective. GFP, RFP and CY5 channels were used for consecutive image acquisition, and z stack images were collected at 5.4-μm steps. Imaging parameters (laser intensity, gain, and pinhole) were kept identical between experiments. NIS Elements software automatically stitched acquired images and converted the stacks into maximum intensity z-projections. Once images were acquired, rostro-caudal level and anatomical location of positive cells were determined using distinctive anatomical landmarks and a mouse brain atlas [23]. Using images taken with a 10x objective, we identified and mapped injection sites by outlining the regions containing transduced cell bodies, independently of density of labeled cells. Regions with fluorescent signals without labeled transduced cell bodies (i.e., areas with labeled processes) were not included in the outlined region. In some cases, more than one outlined region was included per slice to accurately depict the regions containing transduced cells.

Quantification of positive cells

Quantitative analyses were performed between rostro-caudal levels −0.94 and −1.70 relative to bregma for CeA and between −4.96 and −5.34 relative to bregma for PBN. Anatomical delineation for each brain region was determined using a mouse brain atlas [23]. Quantification of positive cells was performed using the NIS Elements software in each channel on one section per rostro-caudal level per mouse. Labeled and co-labeled cells were automatically identified with the NIS Elements software and were visually

confirmed by an experimenter. To quantify the total number of Fos+ and/or mCherry+ cells throughout the entire rostro-caudal PBN, we systematically selected one slice per RC level between -4.96 and -5.34 (relative to bregma) for each animal. The number of positive cells in a total of 4 slices (RC levels: -4.96, -5.02, -5.20, -5.34 relative to bregma) was quantified and the sum was used as the total number of positive cells per brain. All steps in this process were performed blind to treatment.

Ex-vivo **electrophysiology**

Acute CeA and PBN slice preparations

To confirm the effects of clozapine N-oxide (CNO) on hM4Di-transduced cells in the PBN, acute PBN slices were prepared from male C57BL/6J mice from 12 to 18 weeks stereotaxically injected with AAV.hSyn.HI.eGFP-Cre into the CeA and AAV8-hSyn-DIO-hMD4i-mCherry into the PBN. To validate a functional circuit between the PBN and CeA, acute CeA slices were prepared from either *Prkcd*-Cre::Ai9 or *Sst*-Cre::Ai9 male mice (25-26 weeks) injected with rAAV1-hSyn-hChR2(H134R)-EYFP. All electrophysiological experiments were performed at least 4 weeks after stereotaxic injections to allow for efficient uptake of retrograde virus in the PBN (for chemogenetic validation experiments) or efficient expression of ChR2 in PBN terminals in the CeA (for opto-assisted circuit mapping).

Mice were deeply anesthetized with 1.25% Avertin i.p. (0.4 mg/g) and transcardially perfused with an ice-cold cutting solution including: 110 mM choline chloride, 25 mM $NaHCO_3$, 1.25 mM NaH_2PO_4, 2.5 mM KCl, 0.5 mM $CaCl_2$, 25 mM D-glucose, 12.7 mM L-ascorbic acid, and 3.1 mM pyruvic acid. Brains were immediately dissected and submerged in ice-cold cutting solution. Coronal sections including the right CeA (250 μm)

and PBN (300 μm) were cut using feather carbon steel blades (Ted Pella Inc., Redding, CA) and a Leica VT1200 S vibrating blade microtome (Leica Microsystems Inc., Buffalo Grove, IL). CeA and PBN slices were transferred into a recovery chamber of oxygenated artificial cerebrospinal fluid (ACSF) containing 125 mM NaCl, 2.5 mM KCl, 1.25 mM NaH_2PO_4, 25 mM $NaHCO_3$, 2.0 $CaCl_2$, 1.0 $MgCl_2$, and 25 mM D-glucose. Slices recovered for 30 minutes at 33 °C and were then transferred to room temperature for at least 20 minutes prior to recordings. Cutting solution and ACSF were continuously saturated with 95%/5% O_2/CO_2.

Electrophysiological Recordings

Whole-cell, patch-clamp recordings of neurons located in either the PBN or CeA were collected at 33 ± 1 °C. The recording chamber was perfused with oxygenated ACSF (95%/5% O_2/CO_2) at a flow rate of 1 mL/min. An in-line solution heater and heated recording chamber (Warner Instruments) monitored and controlled the temperature conditions throughout the recordings. Neurons were identified through differential interference contrast optics and fluorescent microscopy using an upright telescope (Nikon Eclipse FN1). Recording pipettes (2.5-5 MΩ resistance for CeA and 4-7 MΩ resistance for PBN) were filled with a potassium methyl sulfate-based internal solution (120 mM $KMeSO_4$, 20 mM KCl, 10 mM HEPES, 0.2 mM EGTA, 8 mM NaCl, 4 mM Mg-ATP, 0.3 Tris-GTP, and 14 mM phosphocreatine, with pH adjusted to 7.3 with KOH (approximately 300 mosmol-1). Recordings were performed using a Multiclamp 700B patch-clamp amplifier interfaced with a Digidata 1550 acquisition system and pCLAMP 10.7 software (Molecular Devices) installed on a Dell computer.

Channelrhodopsin-2 (ChR2)-assisted circuit mapping

Optically evoked excitatory postsynaptic currents (oEPSCs) of tdTomato-expressing PKCδ+ or Som+ CeA neurons were recorded at a holding potential of -70 mV, and a single light pulse of 10 ms duration, delivered at 10 Hz, was presented to elicit oEPSCs. Signals were filtered at 10 kHz and acquired at 100 kHz. A blue LED illumination system (λ = 470 nm, Mightex) was used to stimulate PBN-projecting terminals within the CeA. An optical power console and sensor (Thorlabs) measured the blue light output directly through the 40x objective of the microscope prior to each experiment, and the intensity output measured between 10-12 mW. Prior to formation of membrane to pipette seals (~1 GΩ), tip potentials were zeroed. Additionally, the pipette capacitances were compensated and series resistances (not exceeding 20 MΩ) were repeatedly monitored throughout the duration of the recording. Whole-cell capacitance recordings under the voltage-clamp configuration were collected at a holding potential of -70mV and then presented a ± 10 mV voltage change of 25-ms duration. Under current-clamp configuration, 500 ms depolarizing current injections of 220 and 280 pA amplitudes were used to elicit repetitive action potential firing. Cells that categorized as late-firing or regular-spiking based on their latencies to fire in response to prolonged depolarizing current injections, as previously defined [24]. Briefly, regular-spiking (RS) neurons are categorized if the latency to the first spike is shorter than 100 ms and late-firing [25] neurons are categorized if the latency to the first spike is longer than 100 ms. Spike latency for RS and LF cells were assessed in response to 220-pA current step for Som+ neurons and in response to 280-pA current step for PKCδ+ neurons, which elicited an average of 10 spikes in recorded neurons. Current clamp signaling was filtered at 10 kHz

and acquired at 100 kHz. The anatomical location of each CeA neuron recorded was determined using a mouse brain atlas [23].

CeA-projecting PBN neurons recordings and analysis

Animals that received concurrent intra-amygdala injection of Retrobeads™ and sciatic nerve cuff or sham surgery were sacrificed 7 to 10 days after surgery. 300 μm coronal sections containing the right PBN were prepared as described above and allowed to recover in room temperature ACSF for 1 hr prior to recordings. Fluorescently beaded neurons were then targeted for recordings.

Capacitance and input resistance were calculated from a -70 mV ± 10 mV step in voltage clamp. Cells were classified as spontaneous if they fired action potentials while being held at 0 pA. Spontaneous firing frequency was calculated using a 1-minute period of stable firing in current clamp. A series of 500 ms depolarizing current steps (Δ5 pA) were then used to assess firing type and determine the input-frequency relationship. Non-spontaneous (quiescent) cells were classified according to their spike latency to an 80-pA step. Regular spiking neurons had an initial spike latency of less than 20 ms, whereas late firing neurons had an initial spike latency of more than 80 ms. Reluctantly-firing neurons had a rheobase higher than 80 pA, with rheobase defined as the lowest depolarizing current injection amplitude that elicited an action potential. Low-threshold bursting neurons fired a train of action potentials and remained silent after that initial burst across the entire span of current steps (5-200 pA).

Electrophysiological validation of chemogenetic approach

Current-clamp recordings were performed on hM4Di-transduced PBN cells to assess neuronal excitability before and after CNO bath application. All recordings were performed in ACSF with the addition of synaptic blockers (5 μM CPP (-((R)-2-carboxypiperazin-4-yl)-propyl-1-phosphonic acid), 10 μM NBQX (2,3-dioxo-6-nitro-1,2,3,4-tetrahydrobenzo[f]quinoxaline-7-sulfonamide) and 5 μM GABAzine (6-imino-3-(4-methoxyphenyl)-1(6H) pyridazinebutanoic acid hydrobromide). PBN cells expressing mCherry were visually identified and targeted, and series resistances of assessed neurons did not exceed 20 MΩ. To account for the heterogenous firing phenotypes observed within the PBN (i.e. spontaneously active, low threshold, regular-spiking, or late-firing neurons), current clamp protocols were conducted respective to the firing properties. Spontaneously active neurons were recorded continuously for 20 min, while low threshold neurons were recorded using an 800-ms depolarizing current ramp protocol. Action potential firing in regular-spiking or late-firing PBN neurons were recorded in response to a 500-ms square pulse depolarizing current injections. Current injection amplitudes that elicit between 2-5 stable action potentials were used every 15 s. At least 5 stable recordings were obtained prior to bath application of either 10 μM CNO or vehicle (i.e. saline) in ACSF. The number of elicited action potentials during each condition were averaged across 5 traces to assess the effect of CNO on excitability. Current clamp signals were acquired at 100 kHz and filtered at 10 kHz.

Statistical analysis

Data are presented as mean ± SEM. Statistical analysis was performed using unpaired or paired two tailed t test, or two-way analysis of variance (ANOVA) followed by Tukey's

multiple comparison tests using Graph Pad Prism version 9.0. The significance level was set at $p < 0.05$. Sample sizes and p values are described in each figure legend. Detailed information on each statistical test performed are shown in **Table S1**.

1 Wilson TD, Valdivia S, Khan A, Ahn HS, Adke AP, Martinez Gonzalez S, et al. Dual and Opposing Functions of the Central Amygdala in the Modulation of Pain. Cell Rep. 2019;29(2):332-46 e5.

2 Taniguchi H, He M, Wu P, Kim S, Paik R, Sugino K, et al. A resource of Cre driver lines for genetic targeting of GABAergic neurons in cerebral cortex. Neuron. 2011;71(6):995-1013.

3 Hurst JL, West RS. Taming anxiety in laboratory mice. Nat Methods. 2010;7(10):825-6.

4 Carrasquillo Y, Gereau RWt. Hemispheric lateralization of a molecular signal for pain modulation in the amygdala. Mol Pain. 2008;4:24.

5 Ji G, Neugebauer V. Hemispheric lateralization of pain processing by amygdala neurons. J Neurophysiol. 2009;102(4):2253-64.

6 Allen HN, Bobnar HJ, Kolber BJ. Left and right hemispheric lateralization of the amygdala in pain. Prog Neurobiol. 2021;196:101891.

7 Allen HN, Chaudhry S, Hong VM, Lewter LA, Sinha GP, Carrasquillo Y, et al. A Parabrachial-to-Amygdala Circuit That Determines Hemispheric Lateralization of Somatosensory Processing. Biol Psychiatry. 2023;93(4):370-81.

8 Wahlsten D, Hudspeth WJ, Bernhardt K. Implications of genetic variation in mouse brain structure for electrode placement by stereotaxic surgery. J Comp Neurol. 1975;162(4):519-31.

9 Yalcin I, Megat S, Barthas F, Waltisperger E, Kremer M, Salvat E, et al. The sciatic nerve cuffing model of neuropathic pain in mice. J Vis Exp. 2014(89).

10 Benbouzid M, Pallage V, Rajalu M, Waltisperger E, Doridot S, Poisbeau P, et al. Sciatic nerve cuffing in mice: a model of sustained neuropathic pain. Eur J Pain. 2008;12(5):591-9.

11 Carrasquillo Y, Gereau IV RW. Activation of the extracellular signal-regulated kinase in the amygdala modulates pain perception. J Neurosci. 2007;27(7):1543-51.

12 Yoon C, Wook YY, Sik NH, Ho KS, Mo CJ. Behavioral signs of ongoing pain and cold allodynia in a rat model of neuropathic pain. Pain. 1994;59(3):369-76.

13 Colburn RW, Lubin ML, Stone DJ, Jr., Wang Y, Lawrence D, D'Andrea MR, et al. Attenuated cold sensitivity in TRPM8 null mice. Neuron. 2007;54(3):379-86.

14 Hargreaves K, Dubner R, Brown F, Flores C, Joris J. A new and sensitive method for measuring thermal nociception in cutaneous hyperalgesia. Pain. 1988;32(1):77-88.

15 Carrasquillo Y, Gereau RWt. Activation of the extracellular signal-regulated kinase in the amygdala modulates pain perception. J Neurosci. 2007;27(7):1543-51.

16 Randall LO, Selitto JJ. A method for measurement of analgesic activity on inflamed tissue. Arch Int Pharmacodyn Ther. 1957;111(4):409-19.
17 Minett MS, Eijkelkamp N, Wood JN. Significant determinants of mouse pain behaviour. PLoS One. 2014;9(8):e104458.
18 Deuis JR, Dvorakova LS, Vetter I. Methods Used to Evaluate Pain Behaviors in Rodents. Front Mol Neurosci. 2017;10:284.
19 Santos-Nogueira E, Redondo Castro E, Mancuso R, Navarro X. Randall-Selitto test: a new approach for the detection of neuropathic pain after spinal cord injury. J Neurotrauma. 2012;29(5):898-904.
20 Minett MS, Quick K, Wood JN. Behavioral Measures of Pain Thresholds. Curr Protoc Mouse Biol. 2011;1(3):383-412.
21 Anseloni VC, Ennis M, Lidow MS. Optimization of the mechanical nociceptive threshold testing with the Randall-Selitto assay. J Neurosci Methods. 2003;131(1-2):93-7.
22 Costantini I, Ghobril JP, Di Giovanna AP, Allegra Mascaro AL, Silvestri L, Mullenbroich MC, et al. A versatile clearing agent for multi-modal brain imaging. Sci Rep. 2015;5:9808.
23 Franklin KBJ, Paxinos G. Paxinos and Franklin's The mouse brain in stereotaxic coordinates. Fourth edition. ed. Academic Press, an imprint of Elsevier: Amsterdam; 2013.
24 Adke AP, Khan A, Ahn HS, Becker JJ, Wilson TD, Valdivia S, et al. Cell-Type Specificity of Neuronal Excitability and Morphology in the Central Amygdala. eNeuro. 2021;8(1).
25 Woolf CJ, Salter MW. Neuronal plasticity: increasing the gain in pain. Science. 2000;288(5472):1765-9.
